# Adaptive colour change and background choice behaviour in peppered moth caterpillars is mediated by extraocular photoreception

**DOI:** 10.1038/s42003-019-0502-7

**Published:** 2019-08-02

**Authors:** Amy Eacock, Hannah M. Rowland, Arjen E. van’t Hof, Carl J. Yung, Nicola Edmonds, Ilik J. Saccheri

**Affiliations:** 1 Institute of Integrative Biology, University of Liverpool, Liverpool, L69 7ZB UK; 2 Max Planck Institute for Chemical Ecology, Hans-Knöll-Straße 8, Jena, 07745 Germany; 3 Department of Zoology, University of Cambridge, Downing Street, Cambridge, CB2 3EJ UK

**Keywords:** Behavioural ecology, Neurophysiology, Mimicry

## Abstract

Light sensing by tissues distinct from the eye occurs in diverse animal groups, enabling circadian control and phototactic behaviour. Extraocular photoreceptors may also facilitate rapid colour change in cephalopods and lizards, but little is known about the sensory system that mediates slow colour change in arthropods. We previously reported that slow colour change in twig-mimicking caterpillars of the peppered moth (*Biston betularia*) is a response to achromatic and chromatic visual cues. Here we show that the perception of these cues, and the resulting phenotypic responses, does not require ocular vision. Caterpillars with completely obscured ocelli remained capable of enhancing their crypsis by changing colour and choosing to rest on colour-matching twigs. A suite of visual genes, expressed across the larval integument, likely plays a key role in the mechanism. To our knowledge, this is the first evidence that extraocular colour sensing can mediate pigment-based colour change and behaviour in an arthropod.

## Abstract (web summary)

Amy Eacock et al. examine the sensory input mechanism for slow colour change in the twig-mimicking caterpillars of the peppered moth. They find that this camouflage process does not require the use of eyes, relying instead on extraocular colour sensing.

## Introduction

Dermal photoreception, the ability to perceive photic information through the skin independently of eyes, has evolved a number of times to serve a variety of functions^1–4^. It is best known for its involvement in shadow reflexes, phototaxis, and orientation in response to light^5^. More recently, dermal photoreception (more generally referred to as extraocular photoreception) has been proposed to mediate the rapid (physiological) colour change observed in cephalopods^6,7^, fish^8^, and reptiles^9^, through the rearrangement of pigment granules or reflective platelets within specialised cells called chromatophores. Slow (morphological) colour change, occurring over hours to weeks, is common in arthropods^10,11^. Several studies have demonstrated that substrate characteristics^12^ and the wavelength of light^13^ influence pupal colour in a variety of butterfly species^14^, on the assumption that they use their eyes to perceive the colour stimuli. Pioneering experiments by Victorian entomologist Edward Bagnall Poulton on the control of pupal colour in the small tortoiseshell butterfly, *Aglais urticae*, were the first to provide evidence for extraocular photoreception in colour-changing arthropods^15^. Only recently have researchers revisited the possibility that extraocular photoreception is involved in slow colour change of arthopods^16^. Given the prevalence of slow colour change, research is needed to examine the importance of extraocular photoreception in this category of colour change and to characterise the physiological basis of this under-investigated biological phenomenon.

The peppered moth (*Biston betularia*) has evolved to be highly cryptic to visual predators, both in the adult and larval stages. Crypsis is achieved through contrasting mechanisms in each stage. The adult colour pattern polymorphism (melanism) is genetically determined^17,18^, while the larvae camouflage through a combination of twig-mimicking masquerade^19^ and colour plasticity^20^. Colour change in these polyphagous larvae is a continuous reaction norm in response to colour cues from the twigs in the larvae’s immediate surroundings rather than the leaves they eat^20^. The precision of this colour and pattern response is at odds with the simple larval ocelli^21^, and the distal position of the head relative to the twig when larvae are in the resting pose. We conjectured that the larvae could be using an additional visual sense. Here we report the results of morphological, behavioural, and gene expression experiments to investigate the role of extraocular photoreception in colour-changing *B. betularia* larvae.

**Fig. 1:**
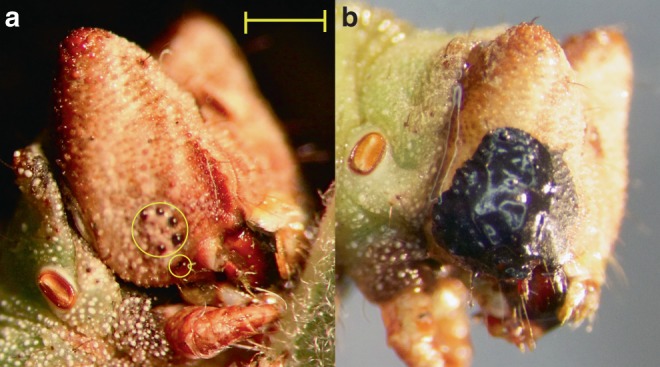
Blindfolding of *B. betularia* larvae. **a** Final (sixth) instar *B. betularia* control caterpillar showing ring of five ocelli circled in yellow, and sixth ventral ocellus circled separately. **b** Example of a final instar larva with ocelli obscured by opaque black acrylic paint. Scale bar represents 1 mm

We reared 321 larvae from 4 families in replicated groups of 25 individuals, inside transparent plastic boxes containing inter-crossing artificial twigs (painted dowels), on stalkless fresh leaves of the grey willow, *Salix cinerea* (see ‘Methods’). We painted over the caterpillars’ ocelli with black acrylic paint with the aid of a microscope (Fig. 1). This obstruction to ocular vision or ‘blindfolding’ started at late second to early third instar, which is the earliest stage at which larvae can be effectively blindfolded, and is prior to a strong colour response. To overcome the problem of caterpillars shedding the blindfold in the process of molting between instars, we checked caterpillars twice daily for early signs of head capsule slippage. Head capsule slippage takes ~12–18 h to complete, during which time we held these individuals separately and singly overnight in opaque white boxes without any dowels. Fresh paint was applied to the new head capsule, thus preventing the caterpillars from receiving any dowel colour signal, and the caterpillars were returned to their group enclosure. We used four different dowel colours, with one colour per enclosure: brown, green, black, and white (see ‘Methods’). The first pair of colours differed in chroma and luminance; the second pair differed only in luminance. The spectral reflectance of each caterpillar’s integument was measured at the final (sixth) instar using a spectrophotometer (six non-overlapping measurements). We used a computational model of visual perception to quantify larval colour and luminance as it would be perceived by a visually hunting avian predator, the blue tit, *Cyanistes caeruleus*^22^. We calculated how green the caterpillars appeared to a predator as the ratio of the medium and long wavelength cone responses; the luminance of each caterpillar as the double dorsal (DD) cone responses; and the discriminability of the larvae as units of ‘just noticeable differences (JND)’ (see ‘Methods’).

## Results

### Colour change

**Fig. 2:**
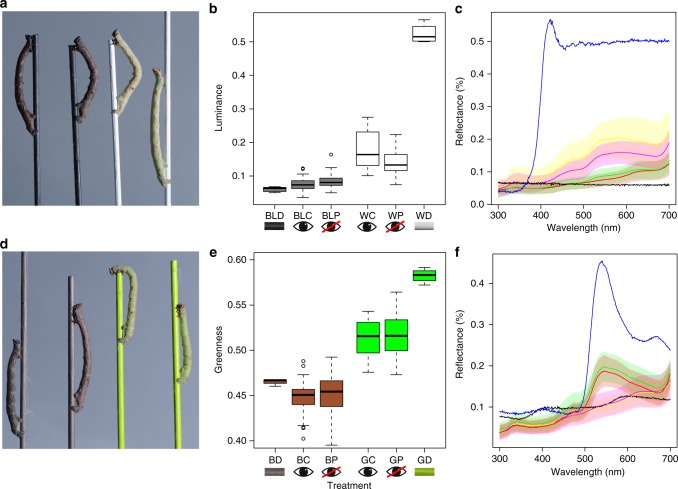
Blindfolded and control *B. betularia* larvae from achromatic and chromatic dowel treatments. **a** Examples of final instar blindfolded (first and third from left) and control (second and fourth from left) larvae on black and white treatment dowels. **b** Luminance of black and white larvae and dowels, calculated from double dorsal blue tit cone catches, where BL = black, W = white, D = dowel, C = control larvae, and P = painted or blindfolded larvae. **c** Reflectance of black and white larvae (mean and standard error) and dowels in the visible wavelength range (300–700 nm, where black = black dowel (BLD), blue = white dowel (WD), red = black control larvae (BLC: *n* = 29), green = black blindfolded larvae (BLP: *n* = 45), yellow = white control larvae (WC: *n* = 26), and magenta = white blindfolded larvae (WP: *n* = 49). **d** Examples of final instar blindfolded (two outermost) and control (two innermost) larvae on brown and green treatment dowels. **e** ‘Greenness’ of brown and green larvae and dowels, calculated as a ratio of mediumwave (MW) to longwave (LW) blue tit cone catches [MW/(MW + LW)], where B = brown, G = green, D = dowel, C = control larvae, and P = painted or blindfolded larvae. **f** Reflectance of brown and green larvae (mean and standard error) and dowels, where black = brown dowel (BD), blue = green dowel (GD), yellow = brown control larvae (BC: *n* = 44), magenta = brown blindfolded larvae (BP: *n* = 50), green = green control larvae (GC: *n* = 36), and red = green blindfolded larvae (GP: *n* = 31). *n* = number of biologically independent samples

We found a striking whole-body colour change in the absence of visual information from the eyes, whereby caterpillars not only changed colour to resemble the dowel colour in their enclosure, but they did so to the same degree as non-blindfolded controls. This is evident to the human eye (Fig. 2a, d), and is also apparent by comparison of the spectral reflectance curves in the visible wavelength range (Fig. 2c, e). However, the more critical and ecologically relevant assessment is through the prism of an avian predator’s perception, which we have quantified through psychophysical modelling. Viewed through this lens, *B. betularia* larvae reared in white dowel enclosures were significantly brighter than those reared on black dowels, when measured as the double cone responses of the avian retina (*F*_1,127_ = 177.4, *P* < 0.0001; Fig. 2b), but there was no significant effect of blindfolding on the luminance of larvae from black or white treatments (*F*_1,127_ = 0.28, *P* = 0.6). Larvae reared in green dowel enclosures were significantly greener to an avian predator than larvae from brown treatments (*F*_1,169_ = 451.2, *P* < 0.0001; Fig. 2e). Moreover, blindfolding had no significant effect on the greenness of larvae in the green or brown treatments (*F*_1,169_ = 0.67, *P* = 0.4), and the distribution of greenness was similar between blindfolded and control larvae across both treatments (Fig. 2e). Using a complementary approach to quantify the ability of an avian predator to distinguish between two stimuli^23^, we find that birds would not be able to discriminate between blindfolded and control larvae, whether reared on achromatic (*F*_1,127_ = 2.64, *P* = 0.1; Supplementary Fig. 1A) or chromatic dowels (*F*_1,169_ = 1.01, *P* = 0.3; Supplementary Figs. 1B and 2).

### Background choice

**Fig. 3:**
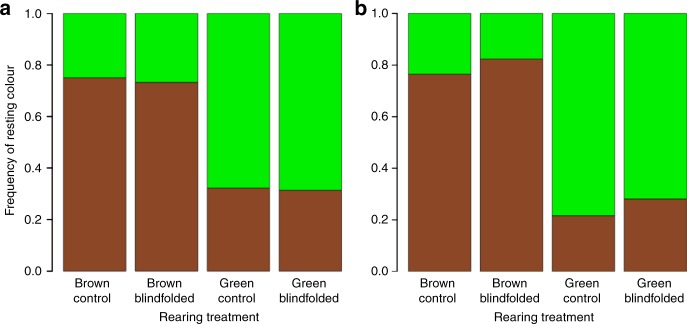
Frequency of resting background colour chosen by *B. betularia* caterpillars. Mean frequency, as proportions of final instar *B. betularia* blindfolded and control caterpillars found on each dowel colour (contrasting luminance green or brown). Individual larvae from blindfolding experiments were placed in either diagonal dowel arenas (**a**), or horizontal dowel arenas (**b**) and their resting choice was recorded after 12 h. Sample sizes (number of biologically independent replicates) are, for crossed dowel and horizontal experiments, respectively: brown control (*n* = 60 and 34), brown blindfolded (*n* = 56 and 34), green control (*n* = 59 and 37), and green blindfolded (*n* = 51 and 32)

To further evaluate the capacity of *B. betularia* caterpillars for extraocular colour perception, we tested background choice behaviour using two designs of background choice arena: a transparent plastic cube containing two diagonally crossing dowels, each painted with a single colour (bright green vs dark brown); and a transparent horizontal tube with a single horizontally suspended dowel, one half painted green, and the other brown (see ‘Methods’). These two designs allowed us to test for the consistency of background choice in different contexts. For each trial, final instar larvae from blindfolded and control groups of the green and brown treatments were placed equidistantly from each dowel colour. Because predation risk increases the likelihood of behavioural background matching, we simulated predation by gently poking larvae on the dorsal surface with tweezers (following methods in reference^24^). For horizontal dowel chambers, to eliminate any positional preferences, two trials were conducted per larva. In one trial, the brown end of the dowel was at the far end of the chamber; in the other trial, the direction of the dowel was reversed (the order of trials was randomised). Individual larvae were left for 12 h (7-h dark, 5-h light), after which the dowel colour that each caterpillar was resting on was recorded. In both types of arena (and both dowel orientations in the horizontal arena), larvae were able to maximise camouflage by selecting dowel colours that more closely matched their own body colours (Fig. 3). On average, 75–80% of brown larvae chose to rest on a brown dowel, and 70–80% of green larvae chose to rest on a green dowel. In the diagonal chamber design, there was no effect of blindfolding (*Z* = −0.22, *P* = 0.83) or larval colour (*Z* = −0.87, *P* = 0.39) on matching success. In the horizontal chamber, there was also no effect of blindfolding (*Z* = −1.24, *P* = 0.21), larval colour (*Z* = 0.82, *P* = 0.41), or dowel position (*Z* = −1.72, *P* = 0.08) on matching success.

### Visual gene expression

**Fig. 4:**
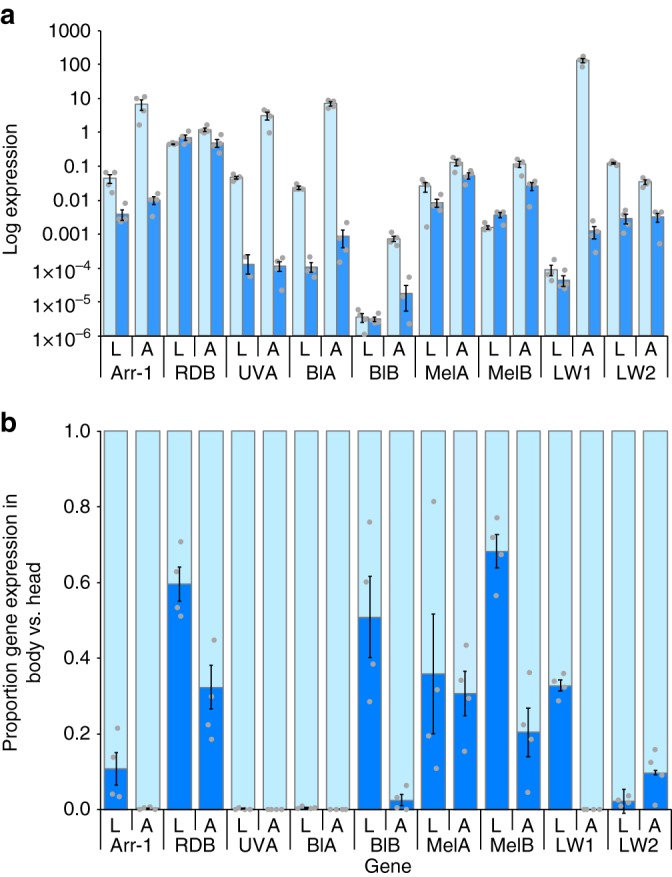
Visual gene expression in head and dermal tissues in larval (L) and adult (A) stages of *B. betularia*. **a** Expression of nine visual gene isoforms relative to a control gene (*spectrin*) in head (light blue) and body tissue (dark blue). **b** Expression of the same visual genes in the skin (dark blue) relative to the head (light blue), calculated as [dermal expression/(head + dermal expression)]. Bars show standard errors (*n* = 4 biologically independent replicates for each stage). Gene names: Arr-1 = arrestin-1, RDB = retinal degeneration B, UVA = ultraviolet wavelength sensitive opsin isoform A, BlA = blue wavelength sensitive opsin isoform A, BlB = blue wavelength sensitive opsin isoform B, MelA = melanopsin isoform A, MelB = melanopsin isoform B, LW1 = long wavelength sensitive opsin copy one, and LW2 = long wavelength sensitive opsin copy two

To investigate the molecular basis of the morphological and behavioural responses, we analysed the expression of key genes involved in visual perception in head (including eyes) and dermal tissue of *B. betularia* larvae and adults. Opsins are light-sensitive proteins that mediate the conversion of a photon of light into an electrochemical signal, necessary for vision and photoreception^25^. We identified opsins sensitive to ultraviolet (two splice variants UVA and UVB), blue (two splice variants BlA and BlB), long wavelength (two gene copies LW1 and LW2), and *melanopsin* (two splice variants MelA and MelB) (Supplementary Figs. 3–5). We also determined the coding sequence (CDS) for visual *arrestin-1* (Arr-1; Supplementary Fig. 6) and *retinal degeneration B* (RDB; Supplementary Fig. 7), which are essential components of phototransduction^26,27^. Using end-point RT-PCR, we detected expression of these genes not only in the eyes (head), but also in all segments of the whole body epidermis, both in larvae and adults (Fig. 4a, Supplementary Fig. 8). Subsequent quantitative assessments using RT-qPCR revealed that in the head tissue, expression levels for several of the genes tested are orders of magnitude higher in adults than in larvae (Fig. 4a; *t*_71_ = −5.33, *P* < 0.0001). This likely reflects the relative size of the compound vs the simple eyes compared to the head of the two life stages. Dermal tissue expression for all genes, averaged across all three body segments, is similar across larvae and adults (Fig. 4a; *t*_69_ = −1.15, *P* = 0.26). Within life stages, dermal expression levels are similar among body segments for most genes (Supplementary Fig. 9). In larvae (Supplementary Fig. 9A), RDB expression is higher in claspers, and BlB expression is much lower in the abdomen; in adults (Supplementary Fig. 9B), RDB expression is lower in the genitalia segment, and UVA expression is somewhat higher in the thorax.

The ratio of gene expression in the epidermis to that in the head provides a measure of the contribution of putative photoreceptors in the larval epidermis to the total light-sensing capacity of a caterpillar. By this measure, dermal expression of photoreception genes is significantly higher in larvae compared to adults (*Z*_11_ = 0.22, *P* < 0.0001), with LW2 as the only gene showing relatively higher dermal expression in adults (Fig. 4b). In larvae, expression of RDB, BlB, and LW1 is upregulated in dermal tissue to similar levels of that in the head. The strongest contrast in relative dermal expression between larvae and adults is for Arr-1, BlB, MelB, and long wavelength copy one (LW1).

## Discussion

*Biston betularia* larvae that were prevented from receiving light input through their ocelli changed colour in response to luminance and colour cues, and also maximised the benefits of this plastic masquerade by actively selecting twigs of similar colour. Experimental and control larvae were equally able to change appearance and choose the appropriate resting background, demonstrating that they are capable of spectrally sensitive extraocular photoreception, and implying that the ocelli play a secondary role in these responses. Our results contrast those of similar blindfolding experiments in other arthropods^10,28^, where the characteristics of the blindfolding paint, rather than the background colour, affected colour change. The necessity for extraocular photoreception in *B. betularia* may relate to the angled twig posture of the larvae during the daytime, which places the ocelli away from the twig perch (Supplementary Fig. 10). In this position, as well as during feeding on leaves, photoreceptors across the larval skin may receive more accurate colour and pattern information about the resting twig than the ocelli.

The potential role of extraocular photoreceptors in colour change via pigment production was first suggested by Poulton^15,29^, working on the determination of pupal colour in *A. urticae*. By means of individual partitioned chambers (i.e., not occluding or destroying the ocelli), in which the head end of a larva and the remainder of the body were exposed to contrasting coloursy, he showed that the resulting pupal colour was determined by the background colour to which the greatest surface area of skin had been exposed. Over one hundred years later, Kato et al.^30^ showed that the pupal greenness of the Japanese oak silkmoth, *Antheraea yamamai*, was determined by the intensity of white light and was unaffected by cauterization of the larval ocelli. Although many other species of caterpillar change colour to better match their resting background^31^, no other research on arthropods has distinguished the role of ocular vs extraocular photoreceptors.

The ability to choose a colour-matching background could be considered redundant in colour-changing animals, such as peppered moth caterpillars, which gain additional protection from predation by masquerading as twigs^32^. However, as colour change in *B. betularia* is a slow process, and the twig colour environment inhabited by these caterpillars is often heterogeneous, background choice matching offers added flexibility and responsiveness. The equivalent strategy of choosing to rest on matching backgrounds in visually heterogeneous environments in species that are also capable of colour change has evolved in flatfish, larval newts, and salamanders^33–35^.

Epidermal opsin expression associated with achromatic light perception has been reported in cnidarians^36^, cephalopods^37^, arthropods^38^, and vertebrates^9^. Given what is known about their primary function, and the energetic cost of gene expression^39^, the relatively high abundance of a whole suite of phototransduction gene transcripts in the larval epidermis suggests that they constitute part of the extraocular photoreceptor machinery. Whether this is also true for the adult moths, which also show appreciable levels of visual gene expression in all segments of their epidermal tissue, is an open question. Precise background matching has been reported for adults of another geometrid moth^40^, but the evidence for *B. betularia*, which occur as a melanic series of genetically determined morphs^41^, is equivocal^42^. To our knowledge, our study provides the first evidence for extraocular opsin expression potentially capable of detecting colour in an arthropod, linked to functional changes in appearance and behaviour.

The identity and precise location of the extraocular photoreceptors remains to be determined. Based on the uniformity and fine grain of the colour change (which is a composite of different epidermal layers; Supplementary Fig. 11), together with the even expression of phototransduction genes across body sections, we speculate that they are distributed more or less evenly within a layer of the larval dermis, rather than in a few spatially restricted specialised cells^38^. Extraocular photoreceptors, resembling light-sensitive *phaosome* cells in earthworm skin, have been described in the genitalia of swallowtail butterflies and proposed to aid in mate choice and oviposition^43^. Whilst the colour response of blindfolded *B. betularia* larvae could, in principle, be produced by a highly compartmentalised physiological mechanism, the background matching behaviour suggests the integration of diffuse information from the epidermis, not only about the twig colours but also resemblance to self. It is therefore likely that the nervous and endocrine systems have a combined role in the colour and background choice responses.

The expression profiles of visual genes in *B. betularia*, combined with morphological and behavioural evidence, lead us to propose that larvae of *B. betularia* possess photoreceptors distributed throughout the epidermis. Their function is to provide more complete information on colour and pattern than can be achieved with the ocelli alone—not only of the resting twig, but also of the match between self and twig. The detailed and composite nature of the caterpillar’s colour pattern suggests a complex signal-processing cascade that initiates, controls, and coordinates the production of multiple pigments in different cell types. Our results significantly expand the current view of dermal light sense to include slow colour change, raising intriguing questions about the evolutionary sequence of pathway recruitment and modification that has culminated in this sophisticated system of extraocular photoreception and phenotypic plasticity, driven by a predator–prey evolutionary arms race.

## Methods

### Dowel experiments

Rearing: To control for any potential genetic effects among families in larval colour responses, the dowel experiments were conducted with a split family design (Supplementary Table 1). *Biston betularia* were reared from eggs and provided with goat willow (*Salix caprea)* ad libitum, with leaves on branches and in the absence of artificial dowels. At second instar, prior to any strong colour-matching response (Supplementary Fig. 12), 25 larvae were transferred to each treatment arena. Treatment arenas comprised of transparent plastic boxes measuring 279 × 159 × 102 mm (length × width × depth) lined with plain blue C-fold 1-ply paper towel, each box containing 20 × 12-cm-long wooden dowels (10 × 5 mm diameter and 10 × 3 mm diameter) held in position by a chicken-wire frame painted to match the colours of the dowels used for each experiment (Supplementary Figs. 13 and 14; Supplementary Table 1). Larvae were fed on *S. caprea* leaves stripped from the branches and stem ad libitum and boxes were washed with 10% bleach every three days to reduce infection risk. Treatment boxes were kept 20 cm apart in a Sanyo Versatile Environment Test Chamber (model MLR-351), with a 12:12 h day:night cycle, at 24 °C in the day with luminescence set at 15,000 lx, and 18 °C at night for the duration of the experiment, until pupation.

Blindfolding: Following a pilot study, black acrylic paint (Royal Langnickel Essentials Acrylic Paint PNTA158 BLACK) was chosen as the most suitable method to occlude light from ocelli and applied using a Royal Langnickel Sable Hair Detail Brush (Liner 5/0,0), with the aid of a microscope. The paint did not permit light transmission (Supplementary Fig. 15). Larvae were checked twice daily for signs of head capsule slippage. Individuals presenting signs of head capsule slippage were removed from the treatment arena and placed into small plastic boxes (70 mm × 70 mm base × 50 mm high) covered in opaque white card, containing only food material (no dowel to rest on). This treatment removed the dowel stimulus whilst maintaining the normal day/night cycle, albeit at a reduced light intensity during the day period. Following complete head capsule slippage, the ocelli of these individuals were re-painted and they were placed back into their designated treatment arenas. The maximum time taken for complete head capsule slippage from beginning to end is 24 h^44^. In this experiment, head capsule slippage was usually completed 6–12 h after larvae were removed from dowels. In this way, there was no point at which the ocelli in the blindfolded group could have received visual information about the dowels. Control larvae were not painted or transferred to isolation boxes. Partial removal of the blindfold was observed only twice out of a total of 11,480 checks across all experiments; these individuals were removed from the experiment.

Quantifying the colour response: Colour quantification and analysis was performed as described in reference^20^. The reflectance of final instar larvae and painted dowels was measured using an Ocean Optics USB2000 spectrophotometer, with a DH-2000 halogen deuterium light source and measured relative to a WS-1 reflectance standard. Larvae were cooled in a fridge for 2–10 min prior to measurement to reduce movement. A total of six measurements were taken: three from the left and three from the right lateral surfaces of each individual, always recorded from the third thoracic segment and the second and sixth abdominal segments. This was to prevent overlap in measurements, and because these segments showed no prominent markings. All spectrometry data were recorded using Overture v.1.0.1.

We processed spectra to 1 nm intervals within the visible light spectrum (300–700) using a program in MATLAB (provided by I. C. Cuthill), and modelled vision in avian colour space using cone photon catches from the blue tit, *Cyanistes caerulus*^22^. Cone stimulation values were converted to Cartesian coordinates and plotted in a tetrahedral space using a custom written MATLAB script^45^, such that each cone is represented by an axis. This colour space is useful because if a colour stimulates only one cone type, then its coordinates lie at the appropriate tip of the tetrahedron, and when all four cone types are equally stimulated its coordinates lie at the origin (Supplementary Fig. 2).

To provide a simpler measure of colour, we calculated greenness as the ratios between the cone catch values of the medium wavelength and long wavelength photoreceptors [MW/(MW + LW)], which represent opponent mechanisms, following Arenas and Stevens^46^. For the black and white dowel experiment we modelled response to luminance but not to colour. We therefore analysed only the blue tit DD cone catch, as these cones mediate luminance vision^22,47^.

We modelled the ease with which an avian predator might discriminate between dowels and larvae using JND; for mathematics, see Vorobyev and Osorio^23^. For chromatic contrasts, we used spectral sensitivities of the blue tit through relative cone ratios of SW = 0.7111; MW = 0.9926; LW = 1.0; and UV = 0.3704^48^, with a Weber fraction of 0.05 and idealised irradiance (D65). To model luminance JNDs, we used blue tit DD cones. JND < 1.00 indicate that two stimuli are indiscriminable; stimuli differing by 1–3 JND units are only discriminable under good viewing conditions; stimuli showing values above this should be distinguishable with increasing ease^49^.

Microhabitat choice: Final instar blindfolded and control larvae that had been reared on brown and green dowels were placed into two designs of choice chamber: one with a choice of two diagonally crossing dowels and one with a single horizontal dowel. The rationale for using two designs was to test larvae under different starting conditions, which may produce initial, non-selective escape responses (onto any twig when the larva is placed on a flat surface). All microhabitat experiments were conducted using 12 individuals at a time in a Sanyo Versatile Environment Test Chamber (model MLR-351) on light level 4 (15,000 lx).

The diagonal habitat choice chamber consisted of a transparent plastic cube measuring 70 × 70 × 80 mm (length × width × depth, including lid) and containing two diagonally crossing 100-mm-long dowels painted in the contrasting colours (brown vs green) that larvae were reared on during blindfolding experiments (Supplementary Fig. 16A). Individual larvae were placed on the base of the diagonal dowel enclosures, equidistantly from each dowel. Prior to placement, larvae were gently poked with tweezers three times along the dorsal surface to simulate predation, as predation risk increases likelihood of microhabitat choice^24^. A sticker with larva ID was placed on the side of each chamber. Individuals were left for 12 h (7-h dark and 5-h light, chosen to reduce disturbance to the natural circadian rhythm of the larvae), after which the dowel colour that each caterpillar was resting on was recorded, followed by the larva ID. One recording was taken per larva.

The horizontal design was a single 200 mm dowel suspended horizontally inside a transparent cylindrical tube measuring 210 × 60 mm (length × diameter) (Supplementary Fig. 16B). Each half of the dowel was painted with the same pairs of contrasting colours as described for the diagonal chamber design. Final instar larvae were subjected to simulated predation and then draped along the centre of the two-tone dowel, not facing either colour. Individuals were left for 12 h (7-h dark and 5-h light), as in the diagonal dowels experiments, and the dowel colour that each caterpillar was resting on and the larva ID were recorded. If the larva position was found to be crossing two colours (<10% of larvae), then the colour that the larva most occupied was recorded. Two experiments were conducted per individual, where the position of the dowel was switched, so that the brown end was facing the base of the chamber (back of the cabinet) for one experiment, and the green end for the other (the order was random). Out of 137 individuals, 34 (~25%) alternated their colour choice between trials.

### Identification and characterisation of visual genes

Visual gene identification: Predicted CDS for ultraviolet (UV) wavelength sensitive opsin, blue wavelength sensitive opsin, melanopsin, and long wavelength sensitive opsin (copy one and two) were obtained (see Supplementary Table 2 for accession numbers) by aligning contiguous sequences from a draft *B. betularia* whole genome sequence (based on NCBI SRA SRX1060178) by tBLASTn^50^ with homologous *Manduca sexta* sequence (Supplementary Table 2), using Geneious, v.5.5.6 (Biomatters Ltd). CDS for RDB and Arr-1 genes were predicted using the same method, with known *Drosophila melanogaster*, *Bombyx mori*, and *Plutella xylostella* homologs (Supplementary Table 2). These CDS were completed and confirmed using a *B. betularia* whole genome BAC library (constructed by Amplicon Express) and a mixture of larval and pupal cDNA from head and dermal tissue. BAC library clones containing sequences of interest were identified from superpools with primers designed from the predicted CDS using Oligo v.6.0^51^ (Supplementary Table 3), and Sanger sequenced (ABI 3130xl).

Phylogenetic analysis: To ensure that visual genes were true homologs, wavelength-sensitive opsins—UV, blue (Bl), LW1, and long wavelength copy 2 (LW2), in addition to Arr-1 and RDB—were aligned with corresponding genes of closely related Lepidoptera species (Supplementary Table 2), obtained using a combination of NCBI BLAST using *B. betularia* sequence as the query sequence. Sequences were aligned manually in MEGA6 v.6.0^52^, and model selection was performed on nucleotide substitutions using the maximum likelihood statistical method for all sites, with complete deletion of gaps/missing data.

Phylogenetic trees for each gene were then constructed from nucleotide substitutions using maximum likelihood. The model used was the best-fitting model based on AICc and BIC values. For UV nucleotide sequences, the best model was the Tamura 3-parameter model with a discrete gamma distribution used to measure evolutionary differences among sites. For Bl and Arr-1 nucleotide sequences, the Tamura 3-parameter model was also used, with a discrete gamma distribution and five rate categories, assuming that a certain fraction of sites are evolutionarily invariable. For LW sequences, the general time reversible model was used, with a discrete gamma distribution and five rate categories, assuming that a certain fraction of sites are evolutionarily invariable. For RDB sequences, the general time reversible model was used, with a discrete gamma distribution. Each phylogeny was constructed using all codon positions and analysis was run using 2000 bootstrap replications. Trees were constructed in MEGA6 v.6.0 and edited in Figtree v.1.4.3^53^.

Gene expression: Four final instar larvae and four imagines (two male and two female) were placed intact (except for gut tissue removal) in 1.5 mL eppendorfs of RNAlater® (Thermofisher) and stored at −80 °C until required. Larvae were later dissected into head, thorax, abdomen, and claspers (Supplementary Fig. 17A; Supplementary Table 1) and imagines were dissected into head, thorax, abdomen, and the distal portion of the abdomen containing the genitalia (Supplementary Fig. 17B; Supplementary Table 1). For all specimens, as much internal tissue as possible was removed from the body, leaving only dermal tissue intact. RNAlater was removed by pipette and the whole tissue was placed in a clean 1.5 mL Eppendorf Safe-Lock Tube containing a 3-mm tungsten bead (Qiagen), to which 1 mL of TRIzol reagent (Thermofisher) was added. Samples were homogenised with a Qiagen tissue lyser II, at 25 Hz for 4 min. Total RNA was isolated following the TRIzol manufacturer’s guidelines (Invitrogen). Genomic DNA was removed from 6 μL of each RNA sample by DNase I Amplification Grade (1 U/μL), following the manufacturer’s protocol. First strand cDNA was synthesised from 5 μL of DNase-treated RNA using 200 U/μL Superscript III Reverse Transcriptase (Thermofisher), following a modified version of the recommended protocol, excluding the RNaseOUT stage and using 0.5 μL of 100 μM Oligo (dT)20 as the anchor primer. Reactions were incubated at 50 °C for 60 min, followed by deactivation at 70 °C for 15 min.

In total we quantified nine visual genes, including splice variants: UV, Bl (splice variants A and B), Mel (splice variants A and B), LW1, LW2, Arr-1, and RDB (Supplementary Fig. 18). We were unable to amplify the alternative splice variant of UV, so only UV splice variant A was quantified. End-point PCR reactions were performed in a Veriti (Applied Biosystems) 96-well thermal cycler with LongAmp® Hot Start Taq DNA Polymerase (New England Biolabs) and the following cycling conditions: 2 min at 94 °C, 40 cycles of [20 s at 94 °C, 30 s at 57 °C, 1 min at 70 °C]. PCR products were loaded onto 2% agarose gel and visualised with 3 μL Midori Green DNA stain (Nippon Genetics) against HyperLadder 50 bp (Bioline). Quantitative PCR was performed using KAPA SYBR fast qPCR (2×) mastermix (KAPA Biosystems), following the manufacturer’s protocol in a final reaction volume of 10 µl containing 0.5 µL cDNA template (diluted to 55%). Each sample measurement was repeated in triplicate and quantified using a Roche Lightcycler 480 II and software v.1.5, under cycling conditions: [3 min at 95 °C, 45 cycles of 3 s at 95 °C, 20 s at optimal annealing temperature, and 20 s at 72 °C]. Melting curves were inspected to ensure single products. Relative expression of PCR product was determined as a ratio against a reference gene, *spectrin* (Supplementary Table 2), which shows uniform expression across cells in *B. betularia*^18^, using [(ERef)^(CpRef)]/[(ETarget)^(CpTarget)]. Here, E = efficiency of PCR reaction (assumed to be the idealised value of 2), Cp = crossing point, Ref = reference gene (*spectrin*), Target = target gene (visual genes). Primers for all PCR reactions were designed using Oligo v.6.0^51^ (see Supplementary Table 3 for sequences).

### Statistics and reproducibility

All statistics were performed using R version 3.3.2^54^.

Colour response: To test whether treatment colour and blindfolding affected the colour and luminance of larvae, as well as their ability to match dowels (JND between larvae and dowels), we fitted linear models to log_10_ JND, greenness, and luminance values. Deviance from normality was checked with qqPlot and by plotting model residuals. Treatment (dowel colour) and blindfolding were tested as predictors of larval luminance (DD) for black and white achromatic treatments, larval greenness for brown and green chromatic treatments, and JNDs for chromatic and achromatic treatments.

Microhabitat choice: To test the effects of blindfolding, treatment, and dowel position (horizontal dowel chambers only) on dowel colour choice, we performed generalised linear models (family = binomial) with larva colour, blindfolding, and dowel position as predictors of matching success (0 or 1).

Gene expression: Comparisons between head and dermal expression of larvae and adults were tested by fitting linear models to the log_10_ of gene expression values. Deviance from normality was checked with qqPlot. To examine the relative contribution of dermal expression within life stages, we used the ratio of dermal to head expression, taking the sum of all the dermal tissue parts (thorax, abdomen, and claspers/genitalia) as a proportion of total expression [dermal expression/(head + dermal expression)]. We modelled this ratio using beta regression, appropriate for proportional data that follows a beta distribution. We tested stage (larva, imago) as predictors of relative dermal expression across genes. Model residuals were checked for normality using qqPlot.

### Reporting summary

Further information on research design is available in the Nature Research Reporting Summary linked to this article.

## Supplementary information

Supplementary Information

Supplementary Data 1

Reporting Summary

Description of additional supplementary files

## Data Availability

Genomic data were submitted to the GenBank database with accession numbers: MH166324–MH166333 (details in Supplementary Table 2). The source data underlying Figs. 2–4 are provided as a source data file. All other data supporting the findings of this study are available from Figshare (10.6084/m9.figshare.8108831) and from the corresponding author upon reasonable request.
