# Supplementary material for: Adaptive colour change and background choice behaviour in peppered moth caterpillars is mediated by extraocular photoreception
Source: Commun Biol. 2019 Aug 2;2:286. doi: 10.1038/s42003-019-0502-7 (PMC6677728; doi:10.1038/s42003-019-0502-7)
Supplement: Supplementary file 1 — Supplementary Information [file 42003_2019_502_MOESM1_ESM.pdf]

Supplementary figures and tables

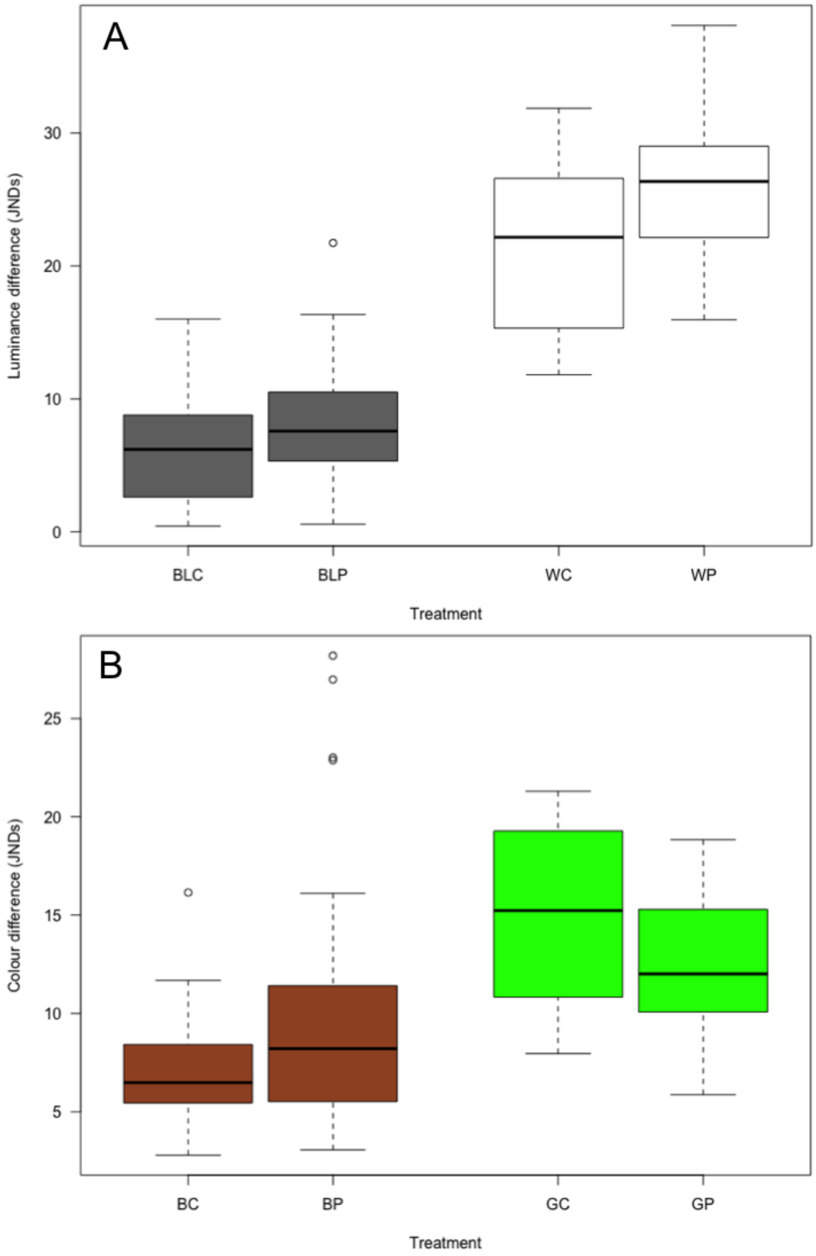

Supplementary Figure 1. Discriminable differences (JND) between *B. betularia* larvae and corresponding dowels, to a blue tit. (A) Luminance differences between black and white larvae from control and blindfolded treatments and corresponding dowels. (B) Colour differences between brown and green larvae from control and blindfolded treatments and corresponding dowels. Treatments are: BLC (black control), BLP (black blindfolded), WC (white control), WP (white blindfolded), BC (brown control), BP (brown blindfolded), GC (green control), and GP (green blindfolded). There was a significant effect of treatment colour on JND for both brown *vs*. green dowels ($F_{1, 169} = 79.14$, $P < 0.0001$) and black *vs*. white dowels ($F_{1, 127} = 165.9$, $P<0.0001$). Blindfolding did not affect JND values for black and white dowels ($F_{1, 127} = 2.64$, $P = 0.1$), or green and brown dowels ($F_{1, 169} = 1.01$, $P = 0.3$).

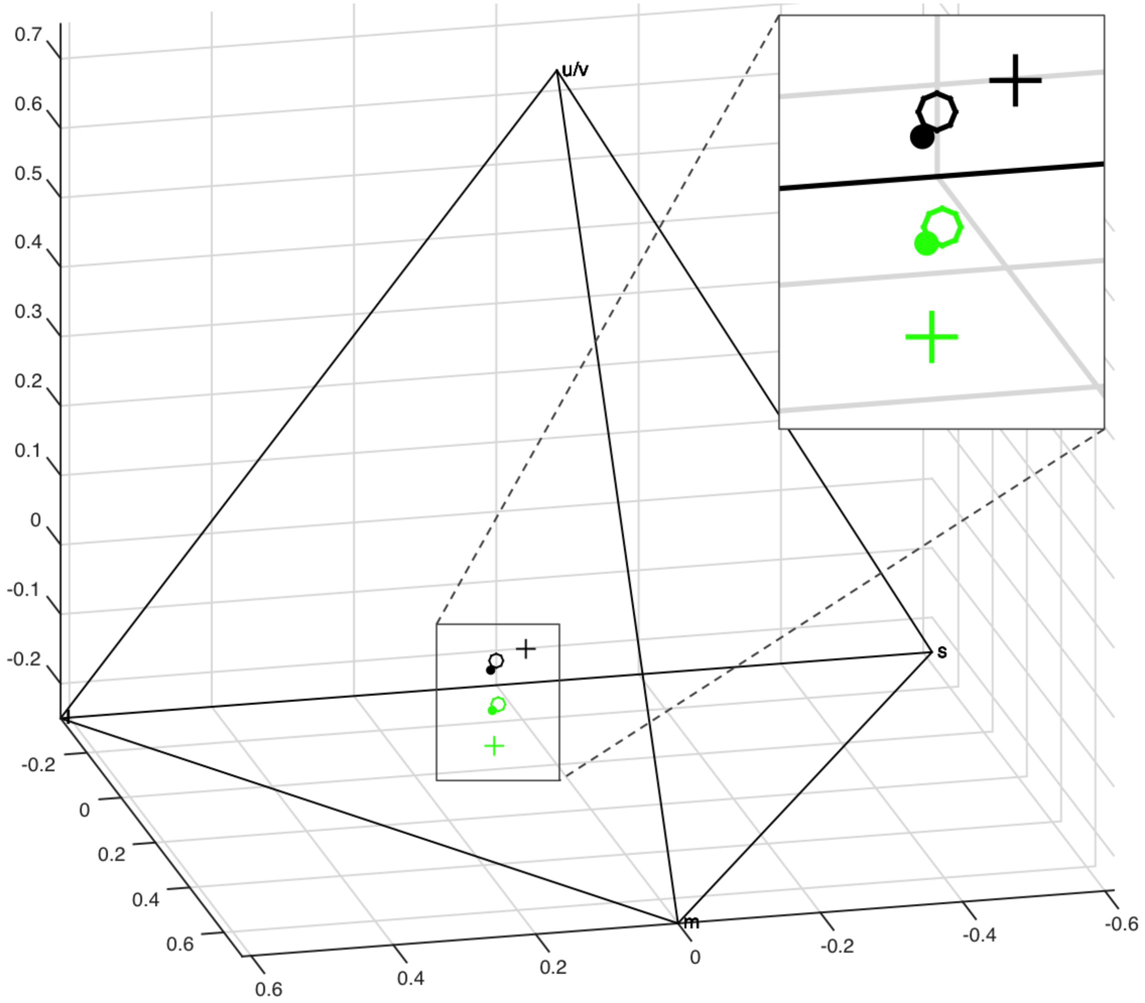

Supplementary Figure 2. The average position of final instar *B. betularia* larvae and their corresponding dowels within the ultraviolet-sensitive (UVS) avian tetrahedral colour space when viewed by a blue tit, *Cyanistes caeruleus*, under bright daylight conditions. Crosses represent dowels; open and closed circles represent larvae from control and blindfolded treatments, respectively; black represents brown treatments and green represents green treatments. The plot illustrates the stimulation of the short (s), medium (m), long (l), and UV (u/v) wavelength-sensitive photoreceptors and is shown from the MW-LW plane.

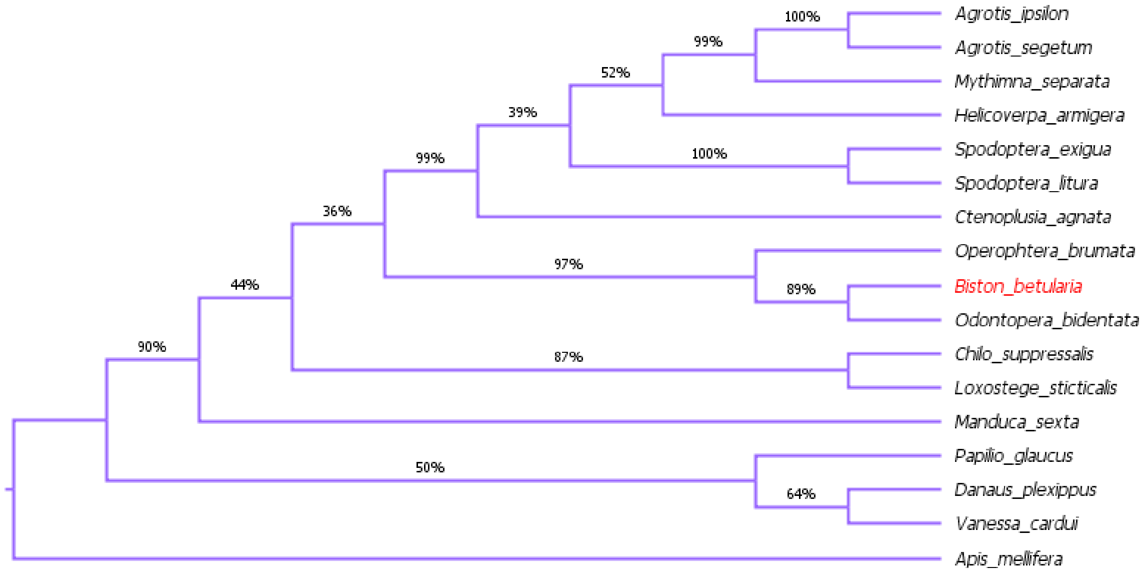

Supplementary Figure 3. Phylogenetic position of *B. betularia* (shown in red) *UV wavelength sensitive opsin* with respect to homologous opsin genes from a sample of 15 other Lepidoptera (Supplementary Table 2), as inferred from ML analysis. Bootstrap percentages based on 2000 replications are displayed on corresponding branches. Cladogram rooted on *Apis mellifera* and evolutionary analyses were conducted in MEGA6.

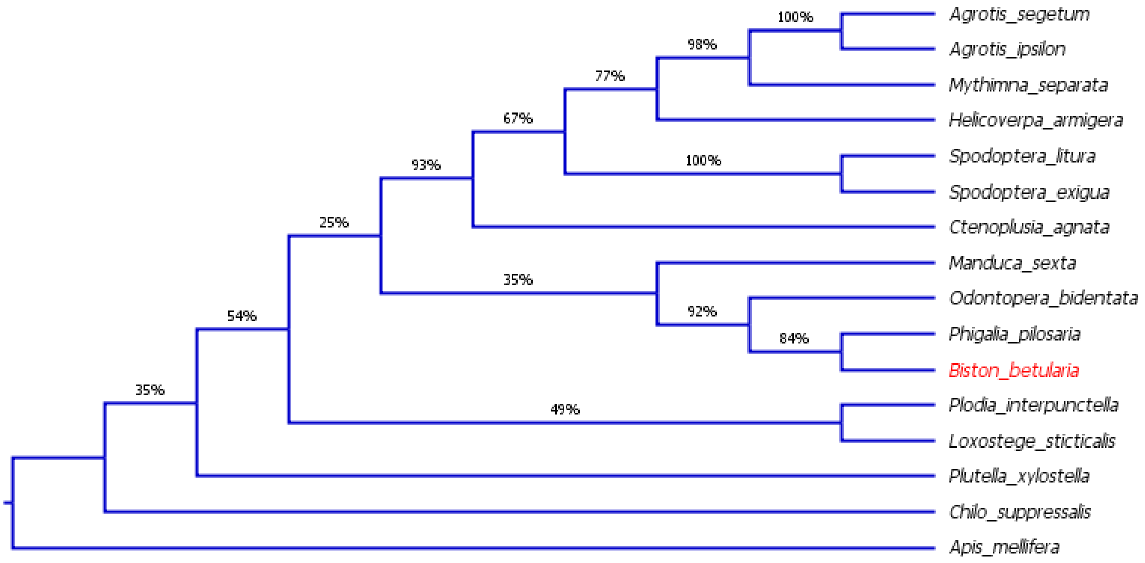

Supplementary Figure 4. Phylogenetic position of *B. betularia* (shown in red) *blue wavelength sensitive opsin* with respect to homologous opsin genes from a sample of 14 other Lepidoptera (Supplementary Table 2), as inferred from ML analysis. Bootstrap percentages based on 2000 replications are displayed on corresponding branches. Cladogram rooted on *Apis mellifera* and evolutionary analyses were conducted in MEGA6.

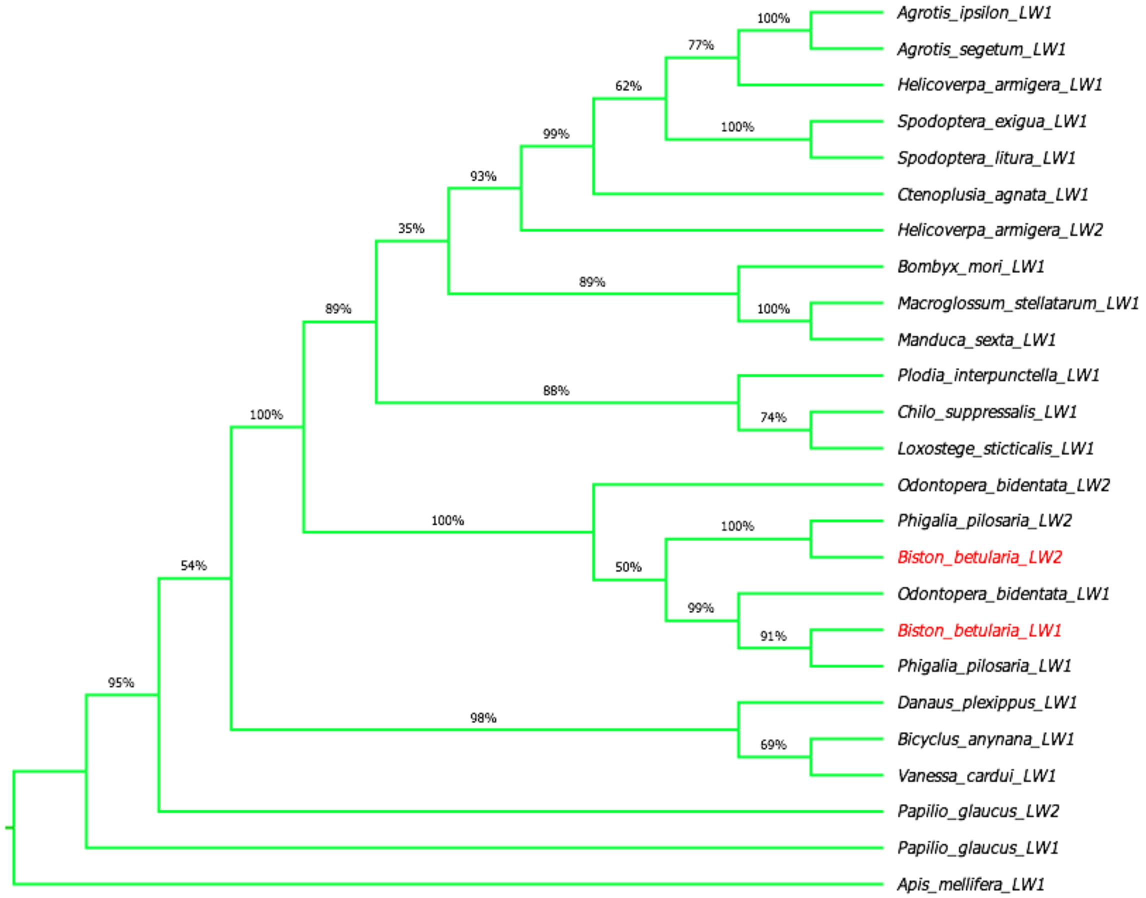

Supplementary Figure 5. Phylogenetic position of *B. betularia* (shown in red) *long wavelength sensitive opsin* with respect to homologous opsin genes from a sample of 23 other Lepidoptera (Supplementary Table 2) as inferred from ML analysis. Bootstrap percentages based on 2000 replications are displayed on corresponding branches. Cladogram rooted on *Apis mellifera* and evolutionary analyses were conducted in MEGA6.

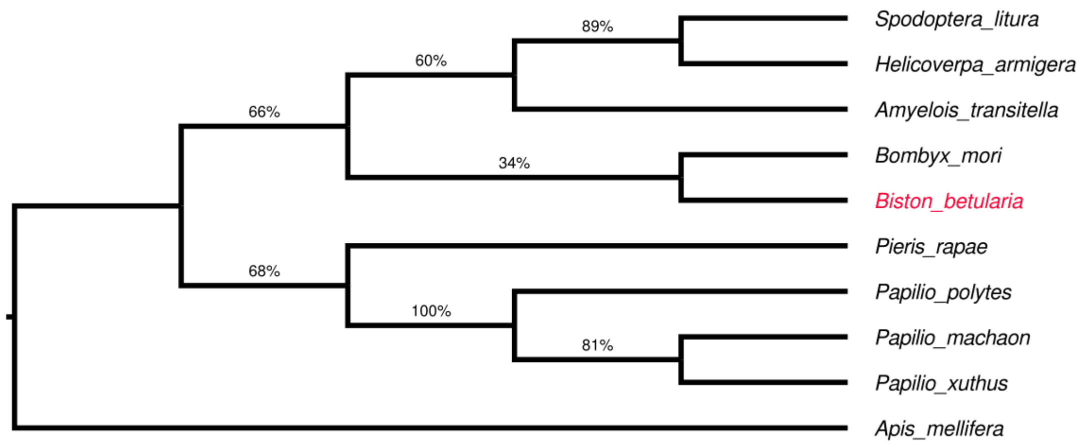

Supplementary Figure 6. Phylogenetic position of *B. betularia* (shown in red) *arrestin-1* (Arr-1) genes with respect to predicted homologous Arr-1 genes from a sample of 9 other Lepidoptera (Supplementary Table 2) as inferred from ML analysis. Bootstrap percentages based on 2000 replications are displayed on corresponding branches. Cladogram rooted on *Apis mellifera* and evolutionary analyses were conducted in MEGA6.

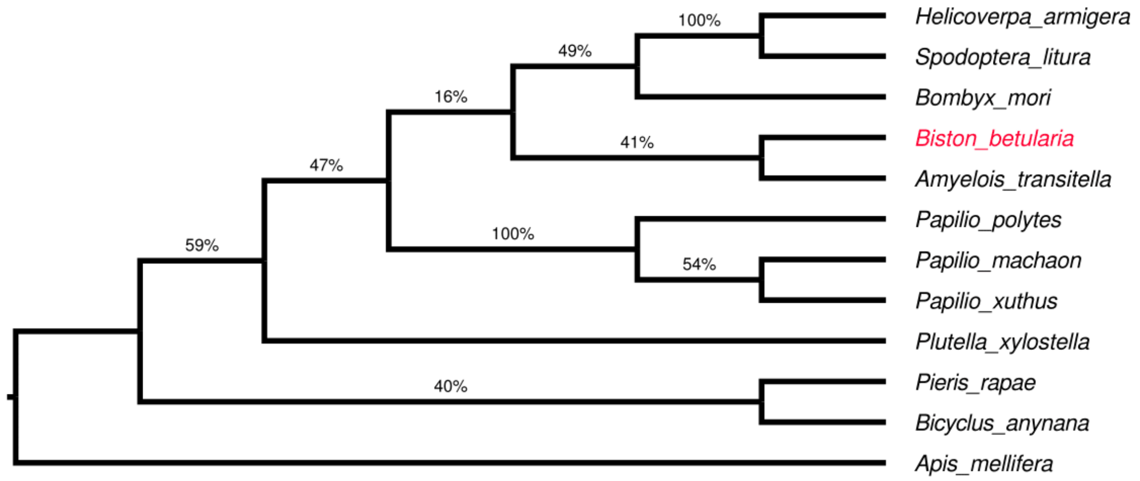

Supplementary Figure 7. Phylogenetic position of *B. betularia* (shown in red) *retinal degeneration B* (RDB) genes with respect to predicted homologous RDB genes from a sample of 11 other Lepidoptera (Supplementary Table 2) as inferred from ML analysis. Bootstrap percentages based on 2000 replications are displayed on corresponding branches. Cladogram rooted on *Apis mellifera* and evolutionary analyses were conducted in MEGA6.

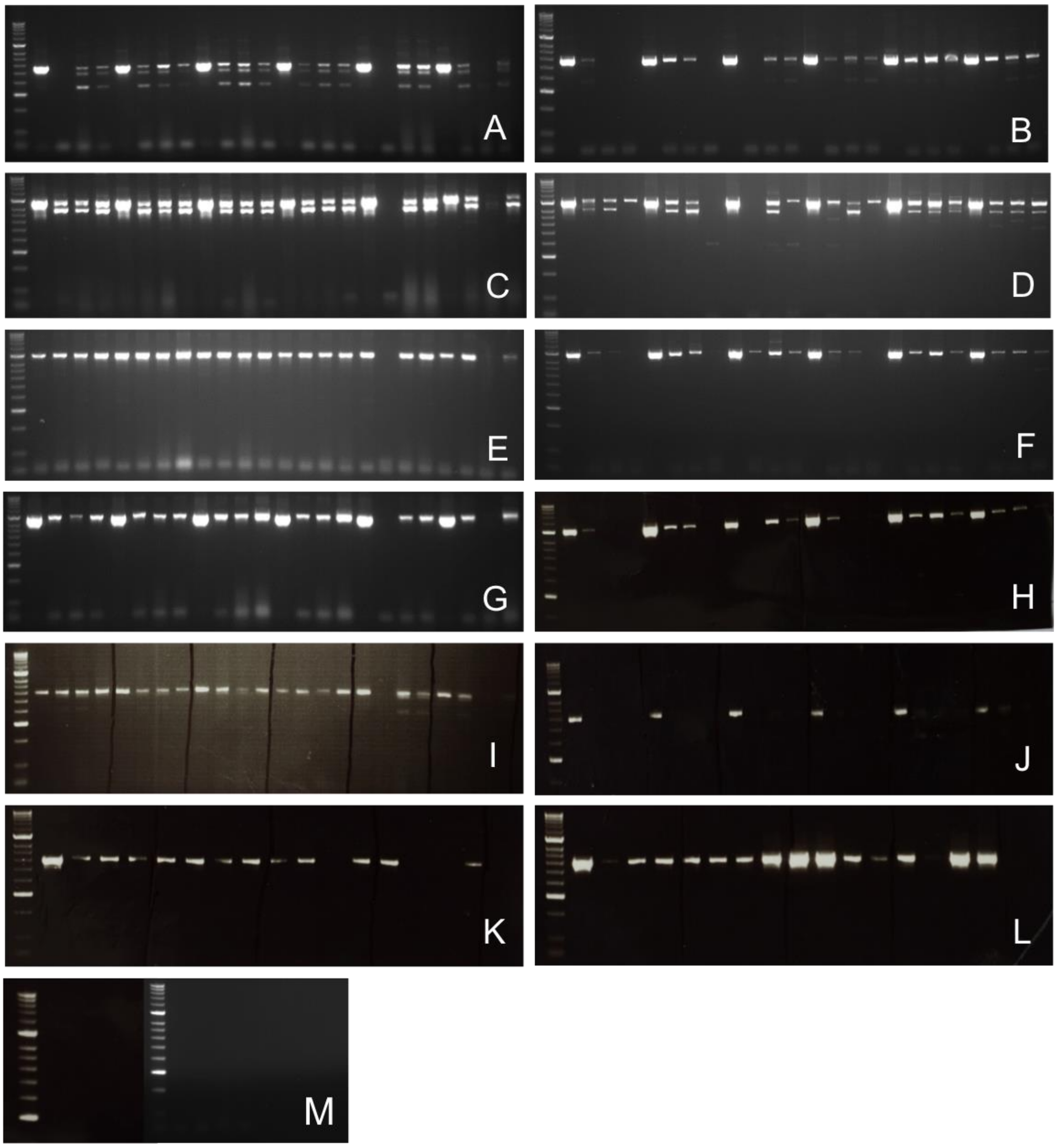

Supplementary Figure 8. End-point PCR gel images showing visual gene expression in tissues of larval and adult stage *Biston betularia*. (A) UV opsin larvae; (B) UV opsin adults; (C) Bl opsin larvae; (D) Bl opsin adults; (E) LW1 opsin larvae; (F) LW1 opsin adults; (G) LW2 opsin larvae; (H) LW2 opsin adults; (I) Arr-1 larvae; (J) Arr-1 adults; (K) RDB larvae; (L) RDB adults; (M) Negative controls. Tissues per individual are shown in multiples of four in the following order: head, thorax, abdomen and claspers (larvae)/genitalia (adults). Six individuals are shown for opsins and *arrestin-1*, and four individuals for RDB. 5µL of PCR product loaded onto 2% agarose gel stained with 1% ethidum bromide, run under 175V, 500A, and visualized under UV light.

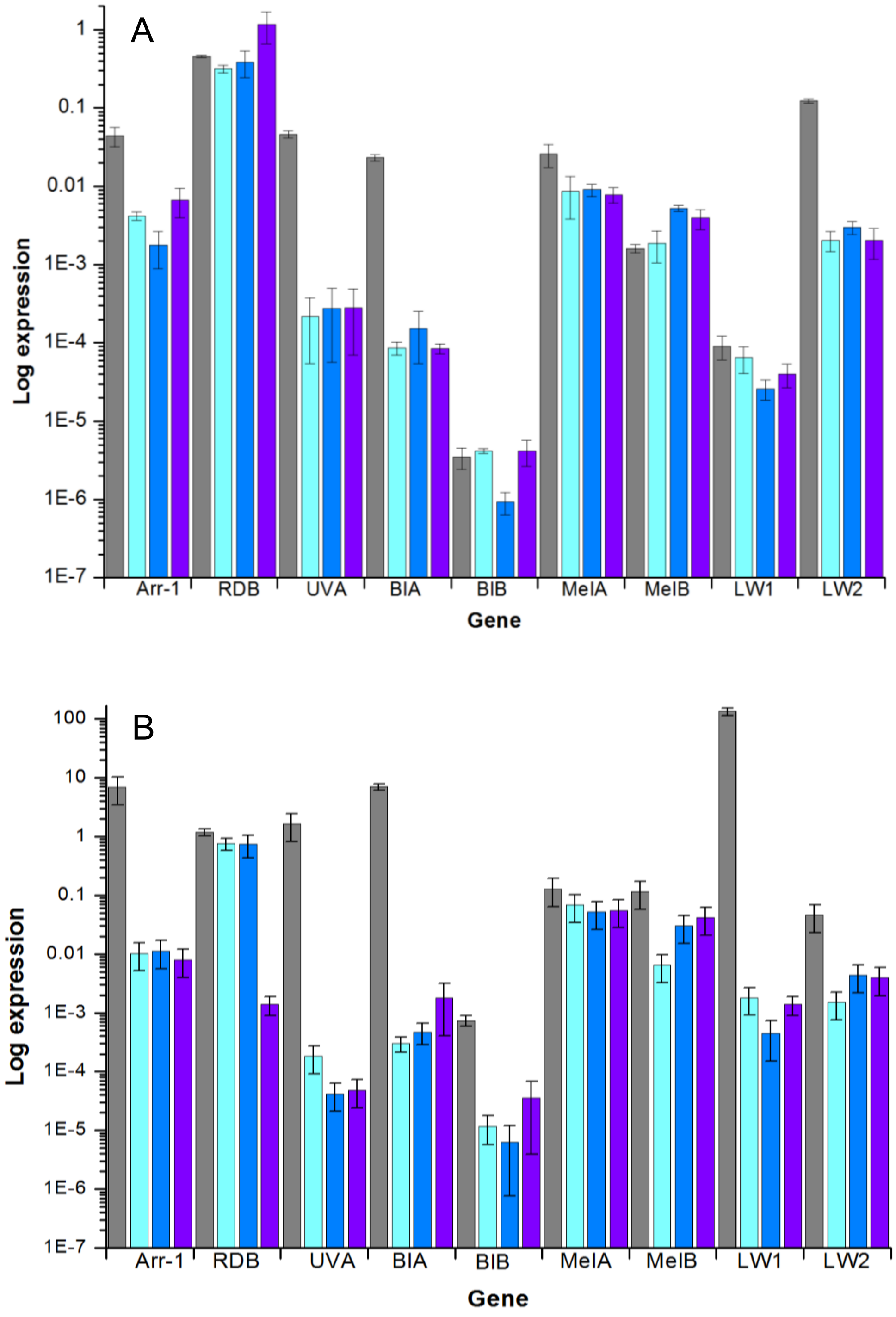

Supplementary Figure 9. Log mean (± SE) relative expression, [(ERef) ^ (CpRef)] / [(ETarget) ^ (CpTarget)], where E = efficiency of PCR reaction and Cp= crossing point, Ref refers to the reference gene *spectrin* and Target refers to each visual gene quantified in head (grey), thorax (cyan), abdomen (blue), claspers, or genitalia in adults (purple) tissue of *Biston betularia* larvae (A), and imagines (B), n=4 biologically independent replicates for each bar.

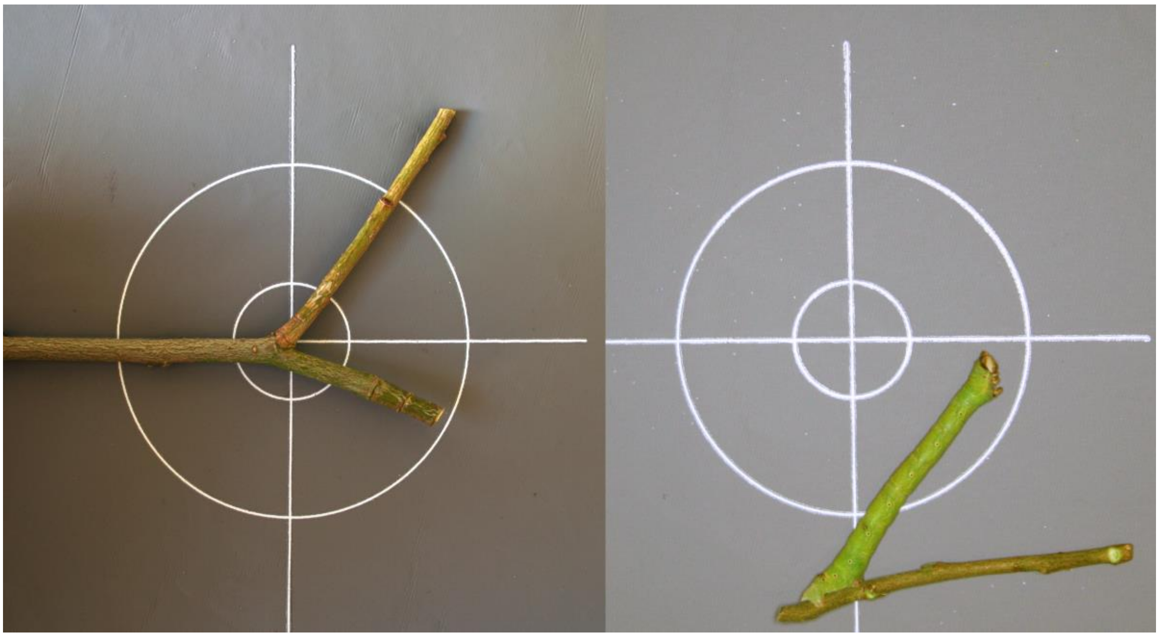

Supplementary Figure 10. The typical resting angle of peppered moth larvae (right) is similar to the angle of a twig on a branch of white willow (*Salix alba*) (left).

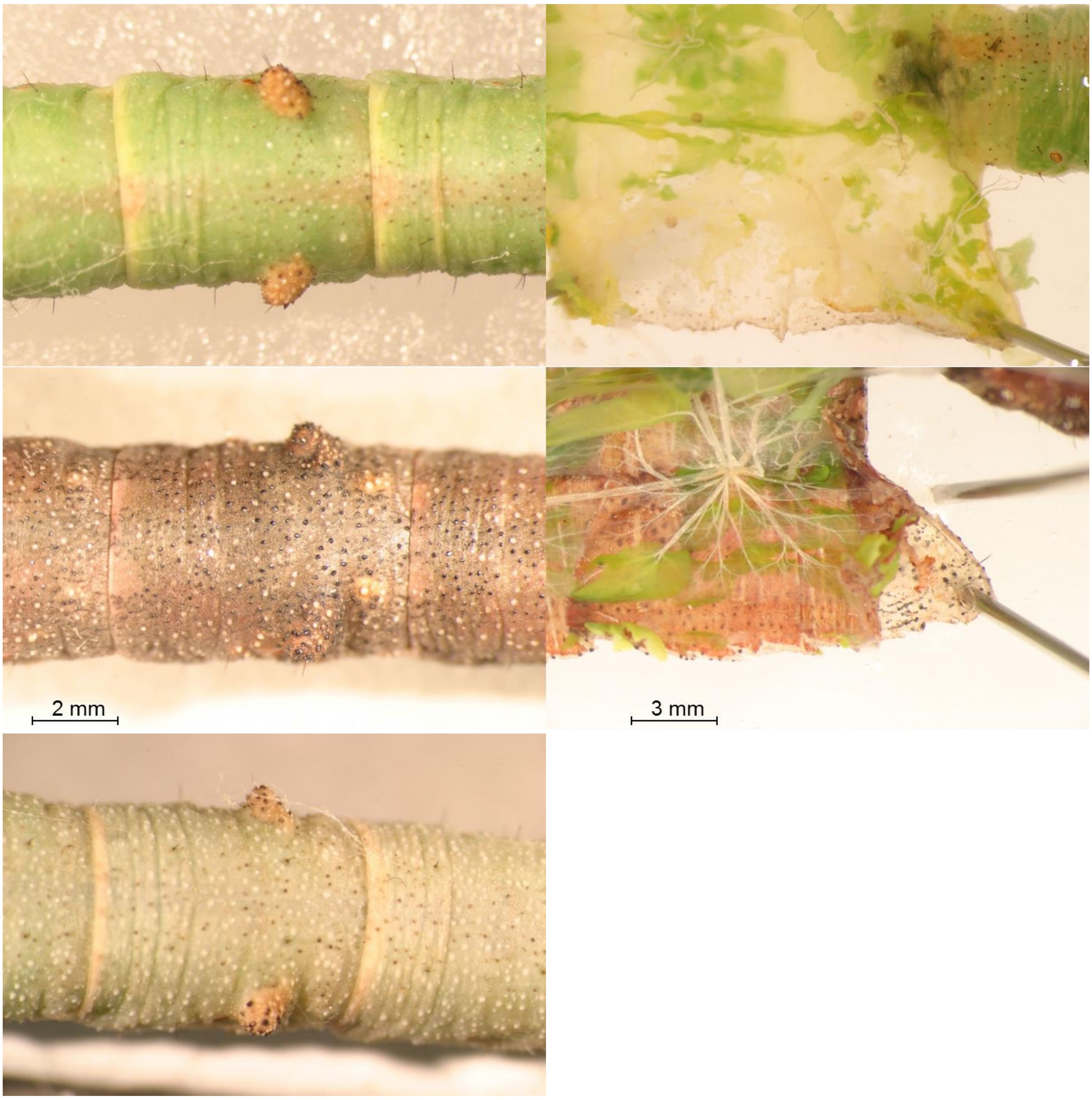

Supplementary Figure 11. Composition of skin colour in green, brown, and 'white' *B. betularia* final instar larvae. The left-hand images show the dorsal exterior surface of abdominal segments 4 to 6 (right to left), with a pair of bumps on segment 5. The right-hand images (green and brown caterpillars only) show a section of the skin viewed from the inner surface, with areas of cream or brown epidermal layer removed. The external colour is a composite of three layers: cuticle, epidermis, and (green) fat. The cuticular layer contains high densities of black and white pimples that can be accentuated (black pimples in brown and black caterpillars, white pimples in 'white' caterpillars) or reduced (black and white pimples in green caterpillars, white pimples in brown caterpillars, black pimples in 'white' caterpillars). In brown caterpillars the epidermal layer gives the background colour, whereas in green caterpillars the cream-coloured epidermal layer is translucent, allowing the bright green fatty tissue to show through. In 'white' caterpillars the cuticular and epidermal layers become whitish and opaque.

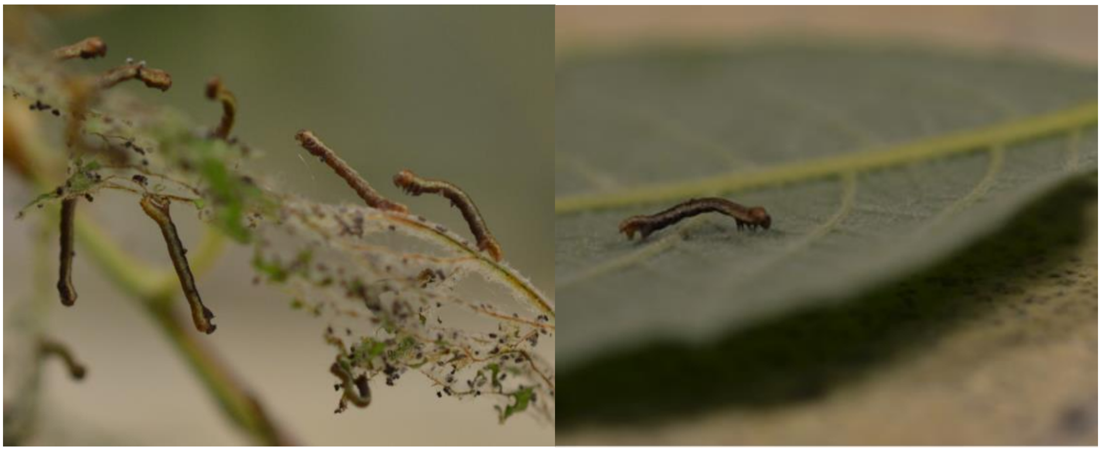

Supplementary Figure 12. Second instar *Biston betularia* larvae ~2 weeks after hatching, showing countershaded appearance. The larva on the right is undergoing head capsule slippage (HCS) before a moult.

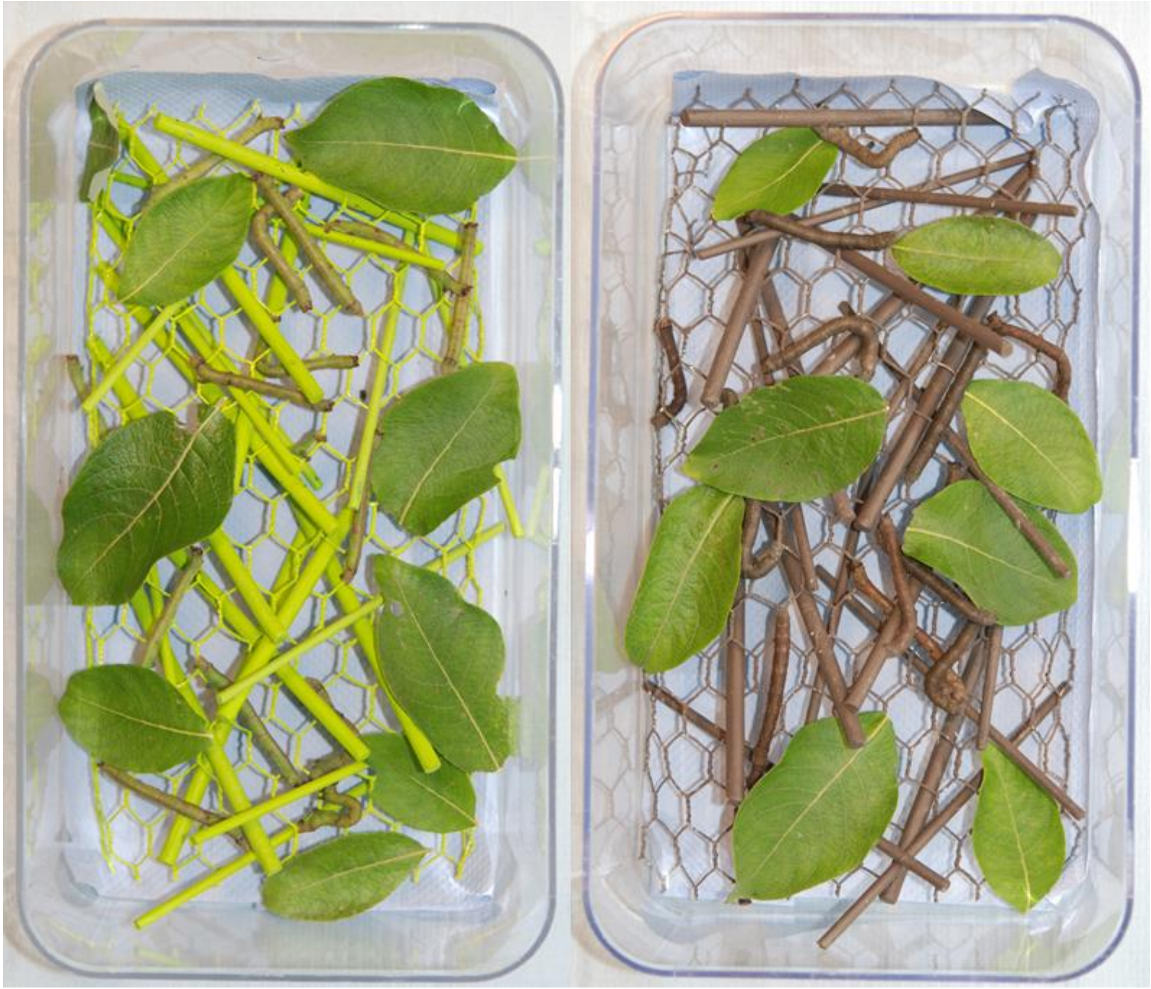

Supplementary Figure 13. Standard treatment set-up used for dowel experiments, showing extreme luminance green (left) and brown (right) dowels, with final instar *B. betularia* larvae.

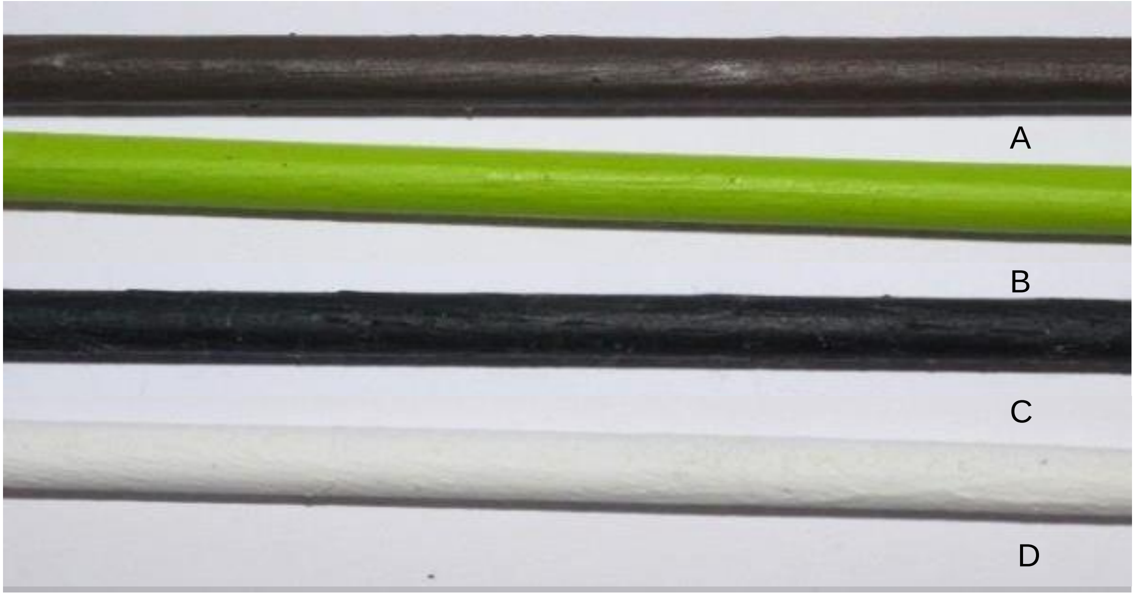

Supplementary Figure 14. All experimental dowel colours with the Dulux paint name parenthesized, A: brown (Espresso shot), B: green (Indian ivy 3), C: black (Night jewels 1), D: white (Chiffon white 4).

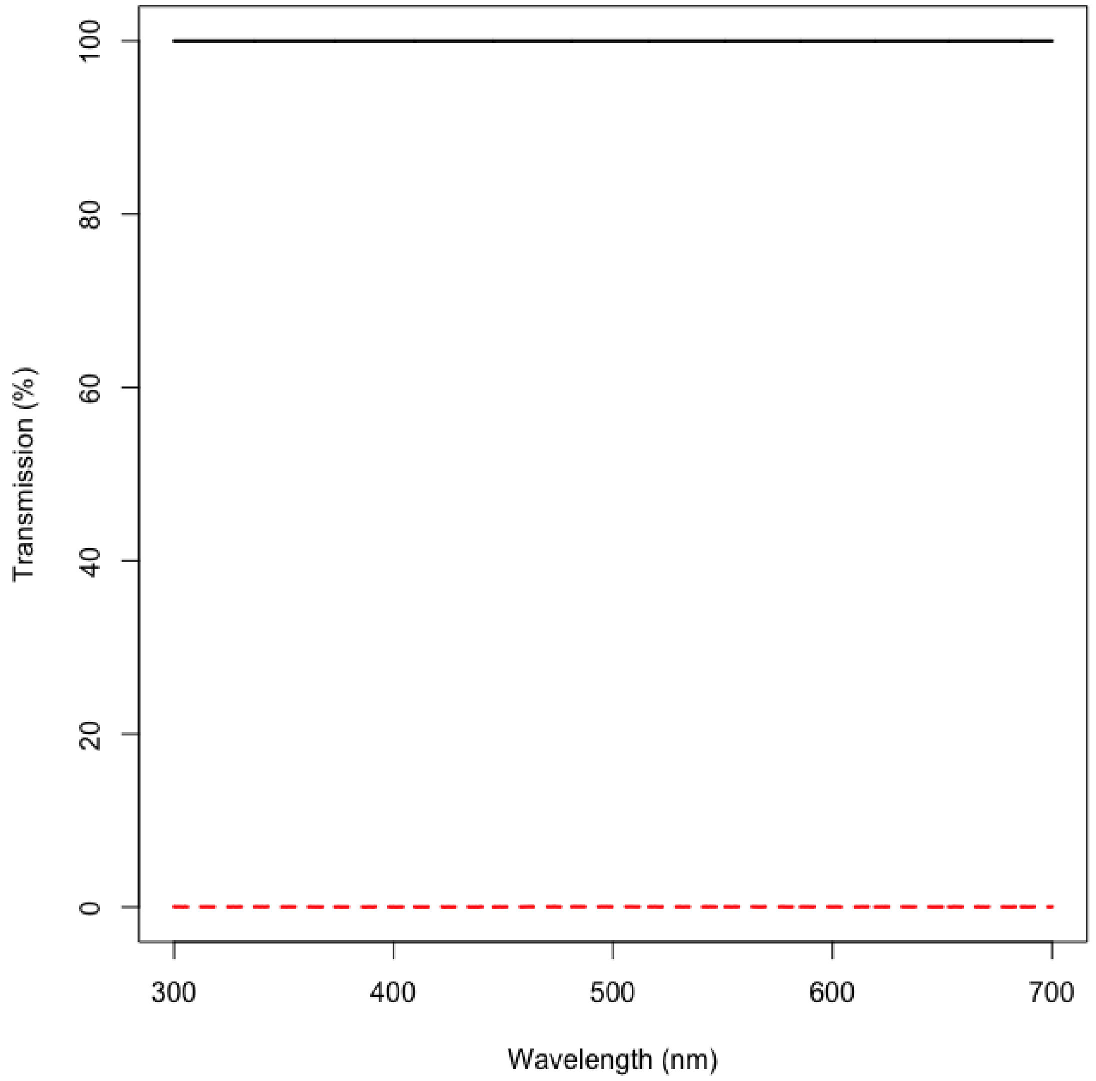

Supplementary Figure 15. Effectiveness of blindfolding at blocking light transmission. Mean percentage of light transmitted through 2mm thick clear plastic when unpainted, representing ocelli of control larvae (black line), and painted with similar thickness of black acrylic paint used to blindfold larvae (dashed red line).

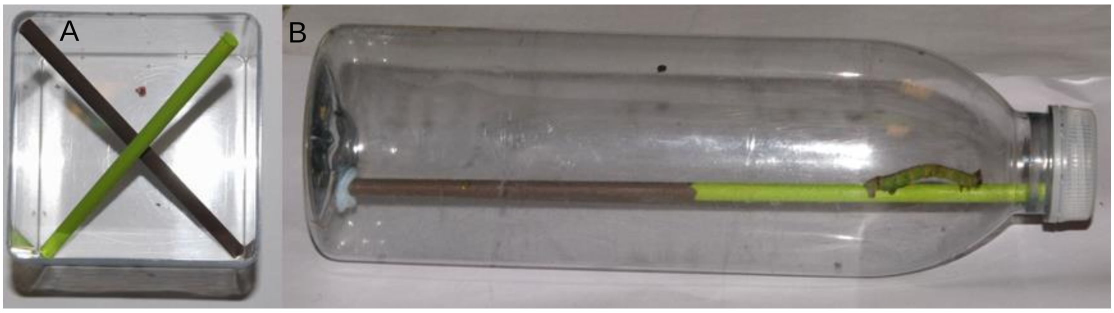

Supplementary Figure 16. Microhabitat choice chamber designs. A) Two diagonally crossed dowel set-up. B) Single horizontal dowel set-up with green treatment *B. betularia* larva.

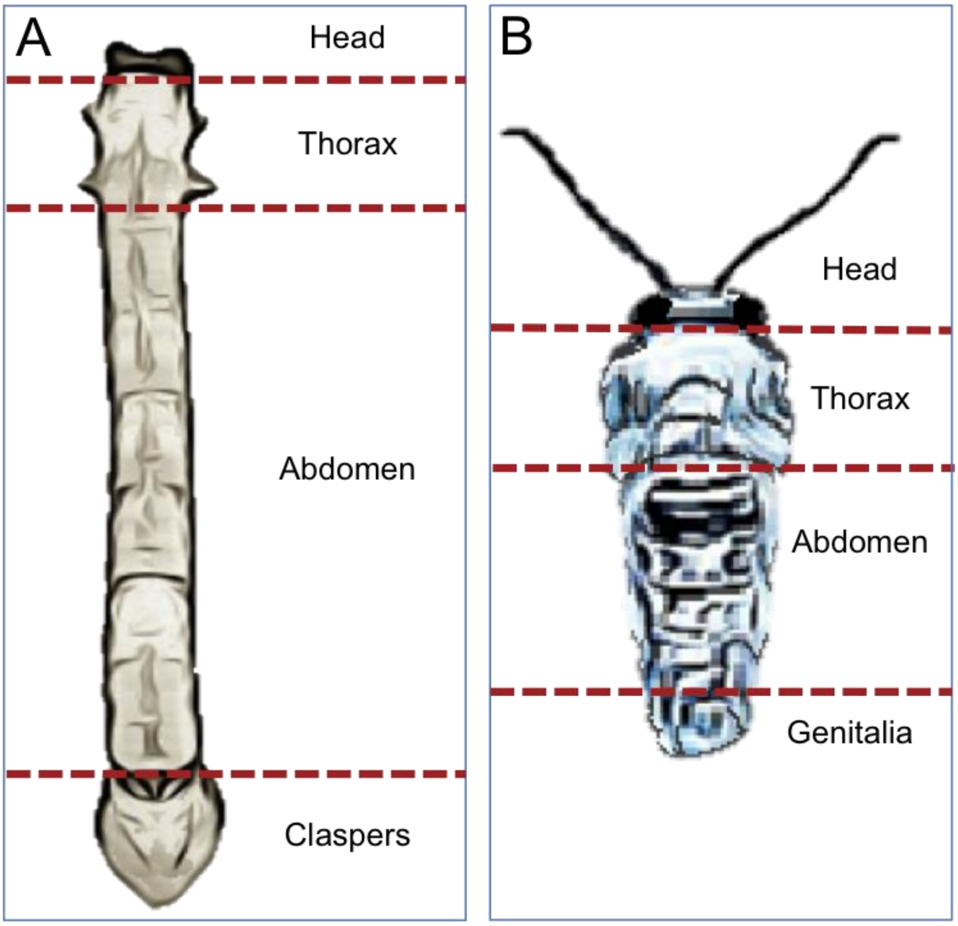

Supplementary Figure 17. Dissection of tissues in *B. betularia*. (A) Four tissue parts of larvae: head, thorax, abdomen, and claspers. (B) Four tissue sections of adults: head, thorax, abdomen, and genitalia. Red dashed line indicates where tissues were separated during dissection.

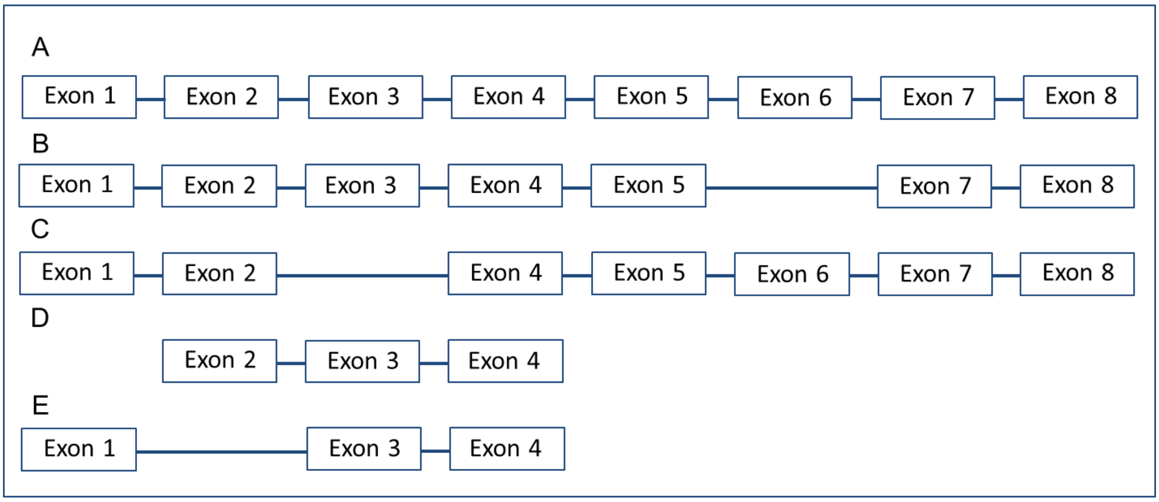

Supplementary Figure 18. Exon-intron structure of opsin genes, including splice variants. A) All eight exons are retained in UV, blue isoform A (BlA), and long wavelength copies one and 2 (LW1 and LW2). B) Blue isoform B (BlB) lacks exon 6. C) UV isoform B (UVB) lacks exon 3. D) *melanopsin* A (MelA) starts at exon 2. E) *melanopsin* B (MelB) skips exon 2.

Supplementary Table 1. Description of *B. betularia* larvae used for all live experiments from 2013-2015, where GC = green control; GP = green blindfolded; BC = brown control; BP = brown blindfolded; BLC = black control; BLC = black blindfolded; WC = white control; WP = white blindfolded. Number after treatment abbreviation indicates replicate box.

Year	Treatment	Dowel paint	Family	Number of replicates (boxes)	Number of individuals per treatment	Total sample size
2013	Blindfolded green	Indian ivy 3	199	3 total – 1x control (GC1); 2x blindfolded (GP1, GP2)	GC1: 10, GP1: 13, GP2: 12	35
2013	Blindfolded brown	Espresso shot	199	3 total – 1x control (BC1), 2x blindfolded (BP1, BP2)	BC1: 15, BP1: 11, BP2: 12	37
2013	Blindfolded black	Night jewels 1	200	4 total – 1x control (BLC1); 3x blindfolded (BLP1-3)	BLC1: 14, BLP1: 14, BLP2: 16, BLP3: 4	48
2013	Blindfolded white	Chiffon white 4	200	4 total – 1x control (WC1); 3x blindfolded (WP1-3)	WC1: 11, WP1: 15, WP2: 15, WP3: 8	49
2013	Opsin expression larvae	-	183	-	4	4
2013	Opsin expression adults	-	183, 164, 186	-	4	4
2014	Blindfolded green	Indian ivy 3	250	4 total – 2x control (GC1, GC2); 2x blindfolded (GP1, GP2)	GC1: 15, GC2: 11, GP1: 14, GP2: 12	52
2014	Blindfolded brown	Espresso shot	250	4 total – 2x control (BC1, BC2); 2x blindfolded (BP1, BP2)	BC1: 15, BC2: 14, BP1: 14, BP2: 13	56
2014	Blindfolded black	Night jewels 1	270	2 total – 1x control (WC2); 1x blindfolded (WP4)	WC2: 12, WP4: 6	18
2014	Blindfolded white	Chiffon white 4	270	2 total- 1x control (BLC2); 1x blindfolded (BLP4)	BLC2: 15, BLP4: 11	26
2014	Microhabitat diagonal	Indian ivy 3, espresso shot	250	8 total – 4x control (GC1, GC2, BC1 BC2); 4x painted (GP1, GP2, BP1, BP2)	GC1: 14, GC2: 12, GP1: 12, GP2: 14, BC1: 15, BC2: 14, BP1: 14, BP2: 11	106
2015	Microhabitat horizontal and diagonal	Indian ivy 3, espresso shot	255	8 total – 4x control (GC1, GC2, BC1 BC2); 4x painted (GP1, GP2, BP1, BP2)	GC1: 20, GC2: 18, GP1: 16, GP2: 17, BC1: 13, BC2: 20, BP1: 17, BP2: 18	139

Supplementary Table 2. Source of visual gene sequences used for phylogenies shown in Supplementary Figures 3-7, including those identified in this study for *Biston betularia*, with Genbank accession numbers where available.

Species	Description	Source	Accession number
***Ultraviolet wavelength sensitive opsin* (UV)**			
Biston betularia	Isoform A	Genome – verified	MH166324
	Isoform B		MH166325
Odontopera bidentata		Genome- predicted	-
Operophtera brumata		Genome – predicted	-
Helicoverpa armigera		NCBI BLAST	HQ641391.1
Agrotis ipsilon		NCBI BLAST	KF539451.1
Agrotis segetum		NCBI BLAST	KF539450.1
Ctenoplusia agnata		NCBI BLAST	KF539452.1
Mythimna separata		NCBI BLAST	KF539458.1
Spodoptera exigua		NCBI BLAST	KF539459.1
Spodoptera litura		NCBI BLAST	KF539460.1
Chilo suppressalis		NCBI BLAST	KF539453.1
Loxostege sticticalis		NCBI BLAST	KF539455.1
Manduca sexta	Manop 2	NCBI BLAST	L78081.1
Papilio glaucus		NCBI BLAST	AF077191.1
Danaus plexippus		NCBI BLAST	AY605546.1
Vanessa cardui		NCBI BLAST	AF414074.2
Apis mellifera		NCBI search	BK005513.1
***Blue wavelength sensitive opsin* (Bl)**			
Biston betularia	Isoform A	Genome- verified	MH166326
	Isoform B		MH166327
Phigalia pilosaria		Genome- predicted	-
Odontopera bidentata		Genome- predicted	-
Helicoverpa armigera		NCBI BLAST	JX644013.1
Agrotis ipsilon		NCBI BLAST	KF539430.1
Agrotis segetum		NCBI BLAST	KF539429.1
Ctenoplusia agnata		NCBI BLAST	KF539431.1
Mythimna separata		NCBI BLAST	KF539428.1
Spodoptera exigua		NCBI BLAST	KF539436.1
Spodoptera litura		NCBI BLAST	KF539437.1
Chilo suppressalis		NCBI BLAST	KF539432.1
Loxostege sticticalis		NCBI BLAST	KF539434.1
Plodia interpunctella		Genome- predicted	-
Plutella xylostella		NCBI BLAST	NM_001305481.1
Manduca sexta	Manop 3	NCBI BLAST	AD001674.1
Apis mellifera		NCBI	BK005512.1
***Long wavelength sensitive opsin* copy one (LW1)**			
Biston betularia		Genome- verified	MH166328
Phigalia pilosaria		Genome- predicted	-
Odontopera bidentata		Genome- predicted	-
Helicoverpa armigera		NCBI BLAST	JX392054.1
Agrotis ipsilon		NCBI BLAST	KF539439.1
Agrotis segetum		NCBI BLAST	KF539438.1
Ctenoplusia agnata		NCBI BLAST	KF539440.1
Spodoptera exigua		NCBI BLAST	KF539448.1
Spodoptera litura		NCBI BLAST	KF539449.1
Chilo suppressalis		NCBI BLAST	KF539441.1
Loxostege sticticalis		NCBI BLAST	KF539443.1
Plodia interpunctella		Genome- predicted	-
Manduca sexta	Manop1	NCBI BLAST	L78080.1
Bombyx mori		NCBI BLAST	XM_021349577.1
Macroglossum stellatarum		NCBI BLAST	KF539444.1
Papilio glaucus		NCBI BLAST	AF077189.1
Danaus plexippus		NCBI BLAST	AY605545.1
Bicyclus anynana		NCBI BLAST	Y918895.2
Vanessa cardui		NCBI BLAST	AF385333.2
***Long wavelength sensitive opsin* copy two (LW2)**			
Biston betularia		Genome- verified	MH166329
Phigalia pilosaria		Genome- predicted	-
Odontopera bidentata		Genome- predicted	-

Helicoverpa armigera		NCBI BLAST	KJ010188.1
Papilio glaucus		NCBI BLAST	AF077190.1
Apis mellifera		NCBI search	NM_001011639.2
***Melanopsin* (Mel)**			
Biston betularia	Isoform A	Genome- verified	MH166330
	Isoform B		MH166331
***Arrestin-1* (Arr-1)**			
Biston betularia		Genome- verified	MH166332
Bombyx mori		NCBI BLAST	XM_004925776.3
Helicoverpa armigera		NCBI BLAST	XM_021342135.1
Spodoptera litura		NCBI BLAST	XM_022965205.1
Amyelois transitella		NCBI BLAST	XM_013338646.1
Papilio machaon		NCBI BLAST	XM_014513530.1
Papilio polytes		NCBI BLAST	XM_013280508.1
Papilio xuthus		NCBI BLAST	XM_013323333.1
Pieris rapae		NCBI BLAST	XM_022262271.1
Apis mellifera		NCBI BLAST	XM_016916562.1
Drosophila melanogaster	Protein for tblastn	NCBI search	NP_476681
Retinal degeneration B			
Biston betularia		Genome- verified	MH166333
Bombyx mori		NCBI BLAST	XM_004929426.3
Helicoverpa armigera		NCBI BLAST	XM_021326572.1
Spodoptera litura		NCBI BLAST	XM_022958969.1
Plutella xylostella		NCBI BLAST	XM_011556250.1
Amyelois transitella		NCBI BLAST	XM_013327659.1
Papilio machaon		NCBI BLAST	XM_014508247.1
Papilio polytes		NCBI BLAST	XM_013287007.1
Papilio xuthus		NCBI BLAST	XM_013324421.1
Pieris rapae		NCBI BLAST	XM_022257986.1
Bicyclus anynana		NCBI BLAST	XM_024080572.1
Apis mellifera		NCBI BLAST	XM_016911166.1
Drosophila melanogaster	Protein for tblastn	NCBI search	NP_476788
**Control gene (*spectrin*)**			
Biston betularia	Reference gene for qPCR	NCBI search	KT182638

Supplementary Table 3. Details of primers used for detecting and sequencing *B. betularia* visual genes.

**Primer(s) used**		**Product length (Bp)**	**Purpose**
***Long wavelength sensitive opsin* copy one (LW1)**			
Bbcon19523r: 1149U<br>AGCTGCCAACCAAACCGTCGTC	Bbcon19523r: 1695L<br>TAAATAACCATGCCGTTGC	526	To confirm exon 1 and 2 sequence in gene copy 1 - superpool
Bbcon03634: 1632U<br>GGGCGTCTTCGAGAGCATGA	Bbcon03634: 2255L<br>AGCGCCTGCTGGTACTTAGGAT	643	To confirm exon 7 and 8 sequence in gene copy 1- superpool
Bbcon19523r: 1184L<br>TCTATCATATGGAGCATATCTG		-	BACs sequencing primer to obtain sequence before exon 1 in gene copy 1
Bbcon03634: 2247U<br>TTCCAGCCATCCTAAGTACC		-	BACs sequencing primer to obtain sequence after exon 8 in gene copy 1
Bb_LW1_1382U<br>ACTAGCGCAGAGTGCAAACTAGC	Bb_LW1_1717L<br>CTGTGGTCGTTCGTCTTGAGATT	358	To target long wavelength copy 1 opsin gene specifically. Used for qPCR temperature gradient
Bb_LW1_519U<br>TGAGAGGTGTATGTTCGCAGGTT	Bb_LW1_747L<br>GATGAAGCCAAGAACGCCGATAG	251	Sequence prediction validation – PCR then sequencing
***Long wavelength sensitive opsin* copy two (LW2)**			
Bbcon08719: 2040U<br>GGTCAAGTGGCCACCTACTCC	Bbcon08719: 2612L<br>AGCCGAGAACACCGATAGTGAA	592	To confirm exon 1 and 2 sequence in gene copy 2- superpool
Bbcon34441r: 377U<br>TACGCTGGAATCTTCGAGAGCA	Bbcon34441r: 811L<br>TAGCGCCTTCTGGTATCTA	454	To confirm exon 7 and 8 sequence in gene copy 2
Bbcon34441: 814U<br>ATACCAGAAGGCGCTATATG		-	BACs sequencing primer to obtain sequence after exon 8 in gene copy 2
Bb_LW2_452U<br>AGAAACCAGTAAACATGTCACCTTCC	Bb_LW2_882L<br>TGGTCCAGATAGAAACGCAACCG	453	PCR and then sequencing to find missing exon 3 and to rectify error within exon 1 from previous *Biston* sequence
Bbcon08719: 2078L<br>CAGCATGTCCGAAGGGACCTTG		-	BACs sequencing primer to obtain sequence before exon 1 in gene copy 2
Bbcon34441r_377U<br>TACGCTGGAATCTTCGAGAGCA	Bb_LW2_1755L<br>TCGCAAGCGGTCGTCATAGTTGT	406	To target long wavelength copy 2 opsin gene specifically. Used for qPCR temperature gradient. Sequencing validation
***Ultraviolet wavelength sensitive opsin* (UV)**			
Bbcon23183r: 555U<br>ACACCGCACTAGCCCTACT	Bbcon23183r: 1077L<br>GTGCGAACACCTGACAACCCAT	542	Verify exon 2 and 3 sequence-superpool
Bbcon128242r: 317U<br>GGCCTCTAGACGGAAGATTATC	Bbcon128242r: 714L<br>AAATGTGTCAAAACCACGTCACTG	417	Verify exon 4 and 5 sequence
Bbcon122456r: 161U<br>ACTTCTCTCTTGGCGTGAAT	Bbcon122456r: 560L<br>GCGAACACTTGTTTCACGATCC	419	Verify contig for exon 5
Bbcon87111: 515U<br>TGGAATCTCTTCGCGCTAATC	Bbcon87111: 911L<br>CGACGATAGTTGTCCGGCACAT	416	Verify contig for exon 7
Bbcon57760: 312U<br>GGGTGTACGCAATCAACCAT	Bbcon57760: 831L<br>GGCAACACTGGTGGTGGTAGAG	359	Verify exon 7 and 8
Bbcon23183r:125L<br>TGCGGTATGCTGCCACTTGTG		-	BACs sequencing primer to obtain exon 1
Bbcon23183r:517L<br>GCCGGGTACGATAGCCAGTGC		-	BACs sequencing primer to obtain exon 2
Bbcon23183r: 1023U<br>AAAGCGCCGATATTCATCTAT		-	Sequencing primer to determine exon 4

Bbcon87111:981U<br>GCACAAGAGTTCGCCTATAC		-	BACs sequencing primer in intron 6 to reach exon 7
Bbcon57760r:290L<br>ACGGATCTATGCAAGACACTAC		-	BACs sequencing primer to determine exon 6
Bb_UVRh_exon7_F<br>CCTGGGTCTACGCCATTAGTCAT		-	BACs sequencing primer
Bbcon57760r: 667U<br>TAAGTCCTCCCTTTGTAAATG		-	BACs sequencing primer to confirm exon 8 and beyond
Bb_UVRh_1000U<br>CGGGATTATCCGATCATCCTGTT	Bbcon23193r_517L<br>GCCGGGTACGATAGCCAGTGC	~277	PCR and sequence to clarify where exon 2 begins
Bb_UVRh_1000U<br>CGGGATTATCCGATCATCCTGTT	Bbcon23183r1077L<br>GTGCGAACACCTGACAACCCAT	~525	PCR and sequence past exon 2
Bb_UVRh_960U<br>CGCTGTGGCGTGTAAAGCTGT	Bb_UVRh_1112L<br>ATTATTTCATGCTGGCGCGTTTG	175	To target UV opsin gene specifically. Used for qPCR temperature gradient
Bb_UVRh_32U<br>GCGCTCATTTTGCAGCTCTCA<br>nested: Bbcon23183r_517L<br>GCCGGGTACGATAGCCAGTGC	Bbcon122456r560L<br>GCGAACACTTGTTTCACGATCC<br>Nested: Bbcon23183r_1077L<br>GTGCGAACACCTGACAACCCAT	709	Sequence prediction validation – PCR then sequencing with nested primers
Bb_UVRh_32U GCGCTCATTTTGCAGCTCTCA	Bbcon23183r_517L<br>GCCGGGTACGATAGCCAGTGC	115	Sequence prediction validation – PCR then sequencing
Bb_UVRh_32U<br>GCGCTCATTTTGCAGCTCTCA	Bbcon23183r1077L<br>GCCGGGTACGATAGCCAGTGC<br>Nested: Bbcon23183r_517L<br>GCCGGGTACGATAGCCAGTGC	363	Sequence prediction validation – PCR then sequencing with nested primers
Bb_UVRh_1000U<br>CGGGATTATCCGATCATCCTGTT	Bbcon122456r560L<br>GCGAACACTTGTTTCACGATCC<br>(nested: Bbcon23183r_517L &1077L)	870	Sequence prediction validation – PCR then sequencing with nested primers
Bb_UVRh_1000U<br>CGGGATTATCCGATCATCCTGTT	Bbcon23193r_517L<br>GCCGGGTACGATAGCCAGTGC	276	Sequence prediction validation – PCR then sequencing
Bbcon12842r317U<br>GGCCTCTAGACGGAAGATTATC<br>Nested: Bb_UVRh_960U<br>CGCTGTGGCGTGTAAAGCTGT	Bb_UVRh_1401L<br>GCAAGCCACAGTCGTCGTTTAACAATAC<br>Nested: Bb_UVRh_1112L<br>ATTATTTCATGCTGGCGCGTTTG	956	Sequence prediction validation – PCR then sequencing with nested primers
Bb_UVRh_375U<br>GGGTTGTCAGGTGTTCGCACTTA	Bb_UVRh_796L<br>TCCGCGCCAGAACTTTGATTAGC	444	To target UV opsin splice variant A specifically. Used for qPCR temperature gradient
Bb_UVRh_238U<br>TTTTCTACACATTCAACAATC	Bb_UVRh_650L<br>CGAGGCAACGAACAGGAAAC	432	To target UV opsin splice variant<br>B specifically. Used for qPCR temperature gradient
***Blue wavelength sensitive opsin* (Bl)**			
Bbcon47669r: 495U<br>CTTCCCGGCCGTTAGCAAGTA	Bbcon47669r: 900L<br>TCCCGCCAAAACTTCCAACTGT	427	Verify exon 2
Bbcon57760r: 313U<br>GGTGTACGCAATCAACCATC	Bbcon57760r: 831L<br>GGCAACACTGGTGGTGGTAGAG	540	Verify exon 7 and 8
Bbcon05474r: 683U<br>GGACGATTTGTGCCAGGTAAAC	Bbcon05474r: 1036L<br>CATTCGCGAGCACAATAC	371	Verify exon 4 to check correspondence of BAC with exon 2 and 8- superpool
Bbcon47669r: 478L<br>GGAAGTTGCGCCAGTGCTCGTG		-	BACs sequencing primer to obtain exon 1
Bbcon57760r: 313U<br>GGTGTACGCAATCAACCATC		-	BACs sequencing primer to obtain 3' UTR
Bb_blueOps_96L<br>CGGGGTTTGATTGATGTTACTAGGT		-	BACs sequencing primer
Bb_blueOps_795U<br>AGCCAACAAGGAAGACGCAAGCA	Bb_blueOps_1090L<br>TGGCAACACTGGTGGTGGTAGA	317	To target blue opsin gene specifically. Used for qPCR temperature gradient
Bb_blueOps_293U<br>TCCGGCACAAGTGAGAAGCGACT<br>Nested: Bbcon47669r: 495U<br>CTTCCCGGCCGTTAGCAAGTA	Bb_blueOps_1021L<br>ATCCCTCTGGCACAAATCGTCCC<br>Nested: Bbcon47669r: 478L<br>GGAAGTTGCGCCAGTGCTCGTG	751	Sequence prediction validation – PCR then sequencing with nested primers

Bb_blueOps_711U<br>AAGCCAAGAAGATGAACGTGAAGT	Bb_blueOps_1134L<br>TCGAACCTTGTTAATGGACCTTGT	447	Sequence prediction validation – PCR then sequencing
Bb_blueOps_728U<br>GCGCTGTCGCTTCTGACG	Bb_blueOps_1090L<br>TGGCAACACTGGTGGTGGTAGA	385	To target blue opsin splice variant B specifically. Used for qPCR temperature gradient
Bb_blueOps_505U<br>GCTGGTCTGCTGATAGCCTTCAC	Bb_blueOps_718L<br>CGTCAGAAGCGACTTTTTTGAACAGTTG	236	To target blue opsin splice variant B specifically. Used for qPCR temperature gradient
***Melanopsin* (Mel)**			
Bb_mopsinX_84U<br>TCGCAGAAGATGCTGGGAGTGCT	Bb_mopsinX_671L<br>TGCTCAGAACTCATCCGCTTATC	610	Superpool and matrix pool for BACs
Bb_mopsinA_186U<br>ACTCCTGGCAACATCCTGATAGC	Bb_mopsinA_306L<br>ACCGCCTACAAATCCATATACGAC	592	Superpool and matrix pool for BACs
Bb_mopsinA_188U<br>TGAGACTTTGGAAGCGACGAAGA	Bb_mopsinA_368L<br>AAACAGCCCATATTTCCGCCACA	203	To target gene melanopsin A specifically. Used for qPCR temperature gradient
Bb_mopsinB_211U<br>TACCGCCTCGCTGAATCTGATGA	Bb_mopsinB_427L<br>CCACTGTAGCGAAGAGTAGATAGAGCAC	244	To target gene melanopsin B specifically. Used for qPCR temperature gradient
Bb_mopsinB_1064U<br>AATAATCAGGCCCATTCAGACGAC	Bb_mopsinA_306L<br>ACCGCCTACAAATCCATATACGAC<br>Nested: Bb_mopsinA_1340L<br>TTCGTATGGTCTCCAGCGGTATA	989	Sequence prediction validation – PCR then sequencing with nested primers
Bb_mopsinB_1169U<br>GTCAAGAGGAACCAATCATCGTAATAAG	Bb_mopsin_1918L<br>GAGCTAGGTTGGCTATCAGGATGTT<br>Nested: Bb_mopsinA_1340L<br>TTCGTATGGTCTCCAGCGGTATA	774	Sequence prediction validation – PCR then sequencing with nested primers
Bb_mopsinA_946U<br>GTGACGTGTAACACATGCGATGTC	Bb_mopsinA_306L<br>ACCGCCTACAAATCCATATACGAC<br>Nested: Bb_mopsinA_1340L<br>TTCGTATGGTCTCCAGCGGTATA	956	Sequence prediction validation – PCR then sequencing with nested primers
***Arrestin-1* (Arr-1)**			
Bb_Arr_68U21<br>AGCGGGACTTCGTCGATCACA	Bb_Arr_619L20<br>ACCGTCTCGCCGTGCATGTA	571	End-point PCR for presence/absence and sequencing
Bb_ArrQ_122U20<br>TGCTGGAGGAGGAGTACGTG	Bb_ArrQ_287L20<br>CCGCTCCTGCGTCTTAGTGA	185	qPCR
***Retinal degeneration B* (RDB)**			
Bb_RDB_113U21<br>GTGGCGTGGAAATTCTTGTCA	Bb_RDB_687L21<br>TGTCTGTGGGCTCGGAGCATA	595	End point PCR for presence/absence and sequencing
Bb_RDB_113U21<br>GTGGCGTGGAAATTCTTGTCA	Bb_RDBQ_EB_429L23<br>CACCATAAAGTTGATCTTTGACT	339	qPCR
**Control genes**			
T7 promotor primer<br>TAATACGACTCACTATAGGG		-	Was used as fosmid forward sequencing primer instead of FosmidF, as provided larger insert
FosmR (fosmidR)<br>CTCGTATGTTGTGTGGAATTGTGAGC		-	Was used as fosmid reverse sequencing primer in BACs sequencing
Bb_RpsA_93U<br>CCGCAAAGACTGGTACGATGT	Bb_RpsA_279L<br>TGCACGTCCTCGGCGATCAAA	207	Control gene for qPCR. Optimised using temperature gradient
Bb_spectrin_278U<br>GCGCTGAAGGAGTTCTCGATGAT	Bb_spectrin_705L<br>TGGAATAGCGTGCGCGTGAAGT	449	Control gene for qPCR.
