# Supplementary material for: Adaptive colour change and background choice behaviour in peppered moth caterpillars is mediated by extraocular photoreception
Source: Commun Biol. 2019 Aug 2;2:286. doi: 10.1038/s42003-019-0502-7 (PMC6677728; doi:10.1038/s42003-019-0502-7)
Supplement: Supplementary file 3 — Reporting Summary [file 42003_2019_502_MOESM3_ESM.pdf]

nature research

Corresponding author(s): Ilik J Saccheri

Last updated by author(s): May 8, 2019

Reporting Summary

Nature Research wishes to improve the reproducibility of the work that we publish. This form provides structure for consistency and transparency in reporting. For further information on Nature Research policies, see Authors & Referees and the Editorial Policy Checklist.

Statistics

For all statistical analyses, confirm that the following items are present in the figure legend, table legend, main text, or Methods section.

n/a	Confirmed	
☐	☒	The exact sample size (*n*) for each experimental group/condition, given as a discrete number and unit of measurement
☐	☒	A statement on whether measurements were taken from distinct samples or whether the same sample was measured repeatedly
☐	☒	The statistical test(s) used AND whether they are one- or two-sided<br>*Only common tests should be described solely by name; describe more complex techniques in the Methods section.*
☐	☒	A description of all covariates tested
☐	☒	A description of any assumptions or corrections, such as tests of normality and adjustment for multiple comparisons
☐	☒	A full description of the statistical parameters including central tendency (e.g. means) or other basic estimates (e.g. regression coefficient) AND variation (e.g. standard deviation) or associated estimates of uncertainty (e.g. confidence intervals)
☐	☒	For null hypothesis testing, the test statistic (e.g. *F*, *t*, *r*) with confidence intervals, effect sizes, degrees of freedom and *P* value noted<br>*Give P values as exact values whenever suitable.*
☒	☐	For Bayesian analysis, information on the choice of priors and Markov chain Monte Carlo settings
☐	☒	For hierarchical and complex designs, identification of the appropriate level for tests and full reporting of outcomes
☒	☐	Estimates of effect sizes (e.g. Cohen's *d*, Pearson's *r*), indicating how they were calculated

Our web collection on statistics for biologists contains articles on many of the points above.

Software and code

Policy information about availability of computer code

Data collection	Raw spectra processing: Overture v.1.0.1.
Data analysis	Bioinformatics: Geneious, v.5.5.6 (Biomatters Ltd).<br>Primer design: Oligo v.6.0<br>Phylogenetic analysis: MEGA6 v.6.0<br>Phylogenetic tree: Figtree v.1.4.3<br>qPCR analysis: Roche Lightcycler 480 II software v.1.5<br>Visual modelling: MATLAB custom code written by I. Cuthill<br>All other statistical analysis: R version 3.3.2

For manuscripts utilizing custom algorithms or software that are central to the research but not yet described in published literature, software must be made available to editors/reviewers. We strongly encourage code deposition in a community repository (e.g. GitHub). See the Nature Research guidelines for submitting code & software for further information.

Data

Policy information about availability of data

All manuscripts must include a data availability statement. This statement should provide the following information, where applicable:

- Accession codes, unique identifiers, or web links for publicly available datasets
- A list of figures that have associated raw data
- A description of any restrictions on data availability

Raw data will be made available on Figshare

Field-specific reporting

Please select the one below that is the best fit for your research. If you are not sure, read the appropriate sections before making your selection.

☐ Life sciences ☐ Behavioural & social sciences ☒ Ecological, evolutionary & environmental sciences

For a reference copy of the document with all sections, see nature.com/documents/nr-reporting-summary-flat.pdf

Ecological, evolutionary & environmental sciences study design

All studies must disclose on these points even when the disclosure is negative.

Study description	For visual gene expression, we quantified 9 visual genes across 4 tissues for two stages (larvae and adult), where biological replicates were n=4 for larvae, and n=4 for adults. For colour change in blindfolded vs. control larvae, 6 replicate colour measurements were taken from each larva and the average was used. A total sample size of 190 blindfolded larvae was compared with 132 non-blindfolded controls, derived from the same families. This was a multifactorial design with box nested in treatment. For the background choice experiments, 119 blindfolded larvae were compared with 116 non-blindfolded larvae. This was a factorial design.
Research sample	The study used peppered moth (Biston betularia) larvae produced from a laboratory stock population established several generations prior to this experiment from wild moths collected from northwest England. Larvae were not sexed, but it was assumed there was an equal ratio of male to female. An equal number of male and female adult moths were used for the gene expression experiment.
Sampling strategy	Sample sizes were chosen to be as large as possible, taking constraints into account.
Data collection	Gene expression data was collected using end-point PCR and qPCR by AE and AVH. Colour data was collected using a spectrophotometer by AE and CY. Behavioural data was collected through observation by AE.
Timing and spatial scale	Due to the life cycle of Biston betularia, experiments were conducted over 3 years, in the summer months of 2013-2015
Data exclusions	Raw spectra from all treatments were examined prior to any colour analyses and any spectra which showed a flat line (i.e., no reflectance values) were excluded as anomalies. This was due to poor calibration from the spectrometer. Only 1-2% of the sample was discarded.
Reproducibility	The colour quantification and behavioural background choice experiments were repeated with numerous treatment boxes across two consecutive years. The gene expression experiments were also repeated, but only the results from the second experiment are shown, as we excluded guts from the adults as well as the larvae. However, despite this change, the results did not significantly differ. Although biological replicates per gene/tissue are <10 (n=4), presentation of mean expression is justified as standard errors are small, and more clearly shows patterns in the data than would individual data points.
Randomization	Individual larvae and adult moths were randomly selected for each experiment.
Blinding	Blinding was used in the behavioural background choice experiments, whereby the treatment ID for larvae was placed on the side of the chamber where it could not be seen until after the larval resting position was recorded.

Did the study involve field work? ☐ Yes ☒ No

Reporting for specific materials, systems and methods

We require information from authors about some types of materials, experimental systems and methods used in many studies. Here, indicate whether each material, system or method listed is relevant to your study. If you are not sure if a list item applies to your research, read the appropriate section before selecting a response.

Materials & experimental systems

n/a	Involved in the study	
☒	☐	Antibodies
☒	☐	Eukaryotic cell lines
☒	☐	Palaeontology
☐	☒	Animals and other organisms
☒	☐	Human research participants
☒	☐	Clinical data

Methods

n/a	Involved in the study	
☒	☐	ChIP-seq
☒	☐	Flow cytometry
☒	☐	MRI-based neuroimaging

Animals and other organisms

Policy information about studies involving animals; ARRIVE guidelines recommended for reporting animal research

Laboratory animals	The study used peppered moth larvae produced from a laboratory stock population established several generations prior to this experiment from wild moths collected from northwest England.
Wild animals	No wild animals were used in the experiments.
Field-collected samples	No field-collected samples were used in the experiments.
Ethics oversight	No ethical approval or guidance was required because the experimental subjects were insects.

Note that full information on the approval of the study protocol must also be provided in the manuscript.
