# Supplementary material for: Adaptive colour change and background choice behaviour in peppered moth caterpillars is mediated by extraocular photoreception
Source: Commun Biol. 2019 Aug 2;2:286. doi: 10.1038/s42003-019-0502-7 (PMC6677728; doi:10.1038/s42003-019-0502-7)
Supplement: Supplementary file 4 — Description of additional supplementary files [file 42003_2019_502_MOESM4_ESM.pdf]

**Description of additional supplementary files**

**File name:** Supplementary Data 1

**Description:** Source data for figures 2-4
